# Identification of novel *mazEF/pemIK* family toxin-antitoxin loci and their distribution in the *Staphylococcus* genus

**DOI:** 10.1038/s41598-017-13857-4

**Published:** 2017-10-18

**Authors:** Michal Bukowski, Karolina Hyz, Monika Janczak, Marcin Hydzik, Grzegorz Dubin, Benedykt Wladyka

**Affiliations:** 1 Department of Analytical Biochemistry, Faculty of Biochemistry, Biophysics and Biotechnology, Jagiellonian University, Krakow, Poland; 2 Malopolska Centre of Biotechnology, Jagiellonian University, Krakow, Poland; 3 Department of Microbiology, Faculty of Biochemistry, Biophysics and Biotechnology, Jagiellonian University, Krakow, Poland

## Abstract

The versatile roles of toxin-antitoxin (TA) systems in bacterial physiology and pathogenesis have been investigated for more than three decades. Diverse TA loci in Bacteria and Archaea have been identified in genome-wide studies. The advent of massive parallel sequencing has substantially expanded the number of known bacterial genomic sequences over the last 5 years. In staphylococci, this has translated into an impressive increase from a few tens to a several thousands of available genomes, which has allowed us for the re-evalution of prior conclusions. In this study, we analysed the distribution of *mazEF*/*pemIK* family TA system operons in available staphylococcal genomes and their prevalence in mobile genetic elements. 10 novel *m* *azEF*/*pemIK* homologues were identified, each with a corresponding toxin that plays a potentially different and undetermined physiological role. A detailed characterisation of these TA systems would be exceptionally useful. Of particular interest are those associated with an SCCmec mobile genetic element (responsible for multidrug resistance transmission) or representing the joint horizontal transfer of TA systems and determinants of vancomycin resistance from enterococci. The involvement of TA systems in maintaining mobile genetic elements and the associations between novel *mazEF/pemIK* loci and those which carry drug resistance genes highlight their potential medical importance.

## Introduction

Toxin-antitoxin (TA) systems are widespread among bacteria, but their physiological roles have only recently been revealed^1–5^. The involvement of these systems in the stress response and the induction of dormancy have been described, and they have also been linked to virulence. However, given the number, variety and broad distribution of TA systems, it is clear that our current understanding only touches the tip of the iceberg.

TA systems were initially described as components of low-copy number plasmids that are involved in the stable propagation of these genetic elements in bacterial populations^6–8^. Because the presence of a long-lived toxin is deleterious, it was thought that cells that are unable to produce a labile antitoxin (i.e. as a result of the loss of the encoding plasmid) would be eliminated. However, the more recent discovery that numerous TA systems are encoded within bacterial chromosomes has sparked a largely unsettled debate regarding their broader role in bacterial physiology^9–13^.

In contrast to our insufficient understanding of the physiological importance of TA systems, the molecular mechanisms of their activity are relatively well understood. In the majority of cases TA systems consist of two components that are ordered within a single operon with the antitoxin gene preceding the toxin gene. Their open reading frames are often partially overlapping. Six types of TA systems have been distinguished based on the nature of the antitoxin (RNA or protein) and its mechanism of action, whereas the toxin component is in all known cases a protein^3,14^. Type II TA systems are composed of protein antitoxin and toxin pairs and are the most prevalent and best-characterised TAs^15^. Under normal conditions, the toxin and antitoxin exist in equilibrium, and the toxin remains dormant. Any disturbance to this equilibrium, regardless of the inducting agent, manifests as a reduction in antitoxin stability, which results in the toxin being unleashed. TA toxins can interfere with important cellular processes, including replication and translation, or induce damage to the cell membrane. There is a significant controversy regarding whether this process reflects a fine-tuned regulatory mechanism or results in unspecific cell “poisoning”. In any case, as a consequence of the toxin’s activity, the cell enters a dormant state. When plasmid loss occurs, the resulting prolonged dormancy leads to cell death^2,16^. However, although rescue mechanisms should exist for the majority of TA systems, in which dormancy is used as an important survival mechanism, the purpose of dormancy remains a matter of lively debate.

The number of TA systems that are encoded in the genome of a particular species of bacteria varies significantly and can range from none (e.g., in *Mycobacterium leprae*) to several dozen (e.g., in *M. tuberculosis*)^17,18^. The rationale for this phenomenon is largely unknown, but more TA systems are carried by virulent pathogens than their non-virulent counterparts, despite the size of their genome, which is usually smaller in virulent species^19^. Despite the significant variety that has been observed among TA systems, certain characteristic features allow bioinformatics to be successfully used to identify novel systems^20–23^. A large, relatively recent study by Makarova *et al*. revealed that there are numerous type II TA systems in prokaryotes^24^. Massive, parallel sequencing has since provided a large amount of genetic data, which prompted us to re-evaluate the distribution and penetrance of TA systems in medically important bacteria in the *Staphylococcus* genus.

Staphylococci are Gram-positive bacteria that are associated with humans and other animals. The majority of species coexist with their hosts as harmless commensals, but *S. aureus* and *S. pseudintermedius* are both dangerous and opportunistic pathogens that are characterised by their increasing drug resistance^25,26^. Current data indicate that staphylococci carry relatively few TA systems. Two paralogues of the YefM/YoeB (*yefM/yoeB*) system were identified in *S. aureus* ^27^. MazEF (*mazEF-Sa*) was found in both *S. aureus* and *S. eqorum* ^10,28^. Moreover, the PemIK (*pemIK-Sa1*) system, which is related to the canonical *mazEF-Sa* system, was identified in a plasmid that was initially isolated form *S. aureus* CH91 and later found in the chromosomes of *S. pseudintermedius* ^12^. These results suggest that this system is mobile. It therefore seems that there are likely to be more such examples in staphylococci.

Here, we analysed currently available staphylococcal genomes to assess the distribution of *mazEF*/*pemIK* family TA system operons and their prevalence in mobile genetic elements. Five computational approaches were evaluated which enabled us to identify 10 novel *mazEF*/*pemIK* systems. A chromosomally encoded *mazEF-Sa* was found in virtually every evaluated staphylococcal genome, suggesting that this element is physiologically important to this group. PemIK systems were less frequent but more diverse. Interestingly, *pemIK* loci were often located in mobile genetic elements in the vicinity of drug resistance genes. Moreover, *pemIK-Sa1* was preferentially identified in poultry-associated strains.

## Results

### General results

**Figure 1:**
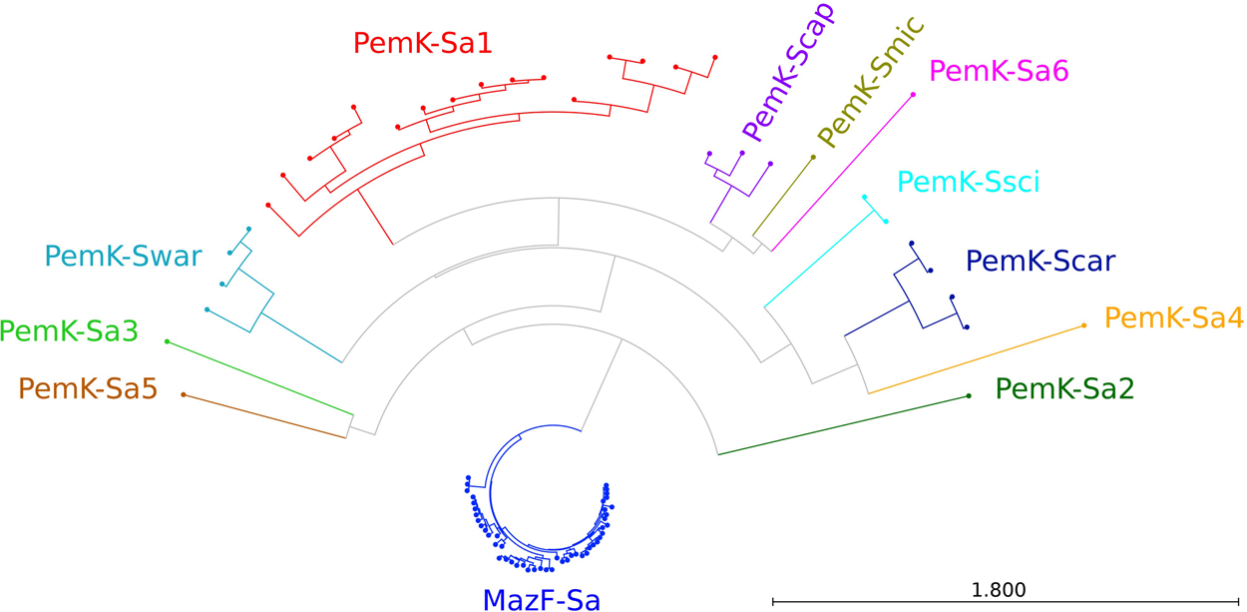
Relatedness of staphylococcal MazF/PemK family toxins. This phylogenetic tree is based on the protein sequences of the toxins. Branches corresponding to the 12 distinguished homologues are marked in different colours. Notably, the MazF-Sa toxin sequences are the least divergent, although *mazEF-Sa* occurs in all staphylococcal strains. By contrast, the PemK-Sa1 toxin sequences for *pemIK-Sa1*, which is the second most frequent TA system of the family in staphylococci, clearly displays a higher degree of divergence.

All 6,132 staphylococcal genomes (representing 36 species) that were published at the time of the study were screened for *mazEF/pemIK* loci. Data collection was substantially biased towards *S. aureus* (5,540 genomes, 90%) because more sequences were available for this clinically significant pathogen. A total of 22,321 open reading frames (ORFs) were identified on average (standard deviation, SD: 2,918) in each genome. Extensive *blastp*, *psiblast*, *rpsblast* and *deltablast* searches resulted in the identification of 73, 83, 101 and 110 two-gene clusters, respectively, and 4, 14, 38 and 41 of these clusters, respectively, were the unique result of a single approach. All clusters were characterised by a high average co-localisation coefficient for member genes (0.65, SD 0.25; 0.69, SD 0.25; 0.70, SD 0.25 and 0.71, SD 0.26; respectively). Remarkably, all four of the approaches returned exactly the same set of *pemIK* homologues (Fig. 1; Suppl. tab. 4). The same results were obtained when tools designed to detect moderate (*blastp*) and distant (*psiblast*, *rpsblast* and *deltablast*) homologues were used, indicating that staphylococcal *mazEF/pemIK* loci comprise a well-defined group of relatively closely related homologues. Among all 12 homologues, only 2 have been described previously, demonstrating the utility of our approach. None of the unique clusters met the exclusion criteria except for several that originated from a DELTA-BLAST search. These contained bicistronic operons that encoded toxins of known families that had never previously been detected in staphylococci, including five Fic/Doc toxins and one each of the following groups: PIN-domain, ParE and Xre toxins. The identification of such distant homologues indicated that our exclusion criteria were sufficiently broad to account for the majority (if not all) of the *mazEF/pemIK* loci in the analysed genomes. PemK-Sa6 is so distantly related to the other analysed proteins that its toxin domain was not detected using *rpsblast*, which relies on direct domain identification within a protein sequence. The operon was, however, identified by all other approaches we used, demonstrating the power of parallel analysis.

### Distribution and genetic context of *mazEF/pemIK* loci

**Figure 2:**
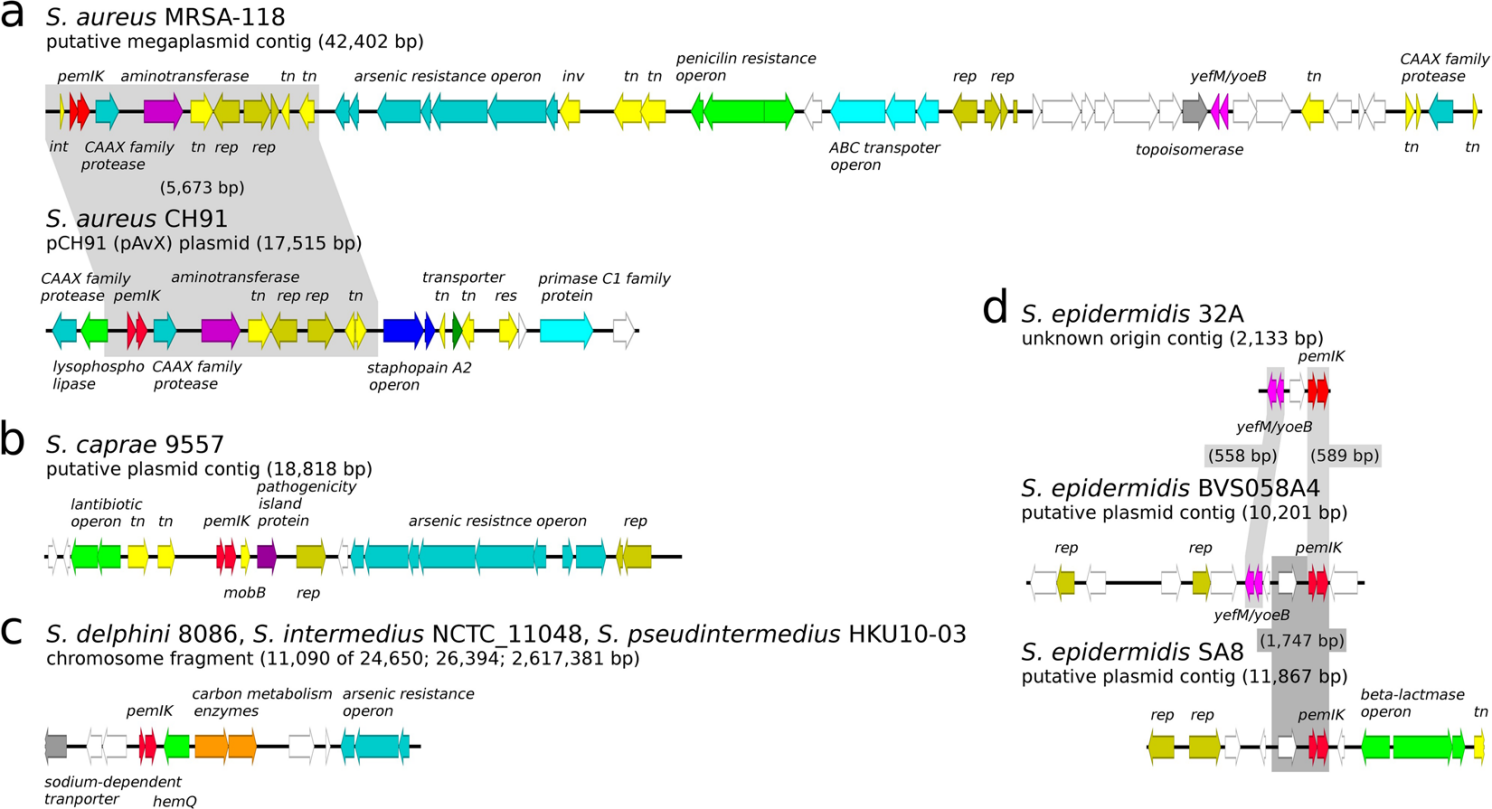
Location of the *pemIK-Sa1* operon within 4 main contexts. The *pemIK-Sa1* loci mainly prevail in plasmids or putative plasmid contigs with the exception of the *S. delphini* – *intermedius* – *pseudintermedius* group, which are located in chromosomes. White arrows indicate sequences that encode hypothetical proteins, and coloured arrows indicate the following: (**a**) in *S. aureus, pemIK-Sa1* is located next to the CAAX family protease, turquoise; aspartate aminotransferase, purple; transposase, transposase fragments and ingerase, *tn*, *int*, yellow; and replication proteins, *rep*, olive. In the *S. aureus* megaplasmid MRSA-118, there were also genes that encoded arsenic resistance, turquoise; penicillin resistance, green a putative ABC transporter, sky blue; and the *yefM/yoeB* toxin-antitoxin system, magenta. The pAvX plasmid also carries the staphopain A2 operon, blue and the primase C1 family protein, sky blue. (**b**) In this context, represented by a putative contig that was derived from *S. caprae* 9557, *pemIK-Sa1* is in the neighbourhood of genes that encode the following: antibiotic resistance, green; transposase, *tn*, yellow; protein involved in plasmid mobilisation, *mobB*, yellow; replication proteins, *rep*, olive; and arsenic resistance, turquoise. (**c**) In the *S. delphini* – *intermedius* – *pseudintermedius* group, *pemIK-Sa*1 is located in proximity to genes that encode the following: a sodium transporter, grey; a haemoprotein involved in heme biosynthesis, *hemQ* green; the phosphate acetyltransferase lipoate-protein ligase A, orange; and arsenic resistance, turquoise. In the last context shown (**d**), the operon in the neighbouring genes encodes replication proteins, *rep*, olive; the *yefM/yoeB* toxin-antitoxin system, magenta; beta-lactams resistance, green; and transposase, *tn*, yellow.

**Figure 3:**
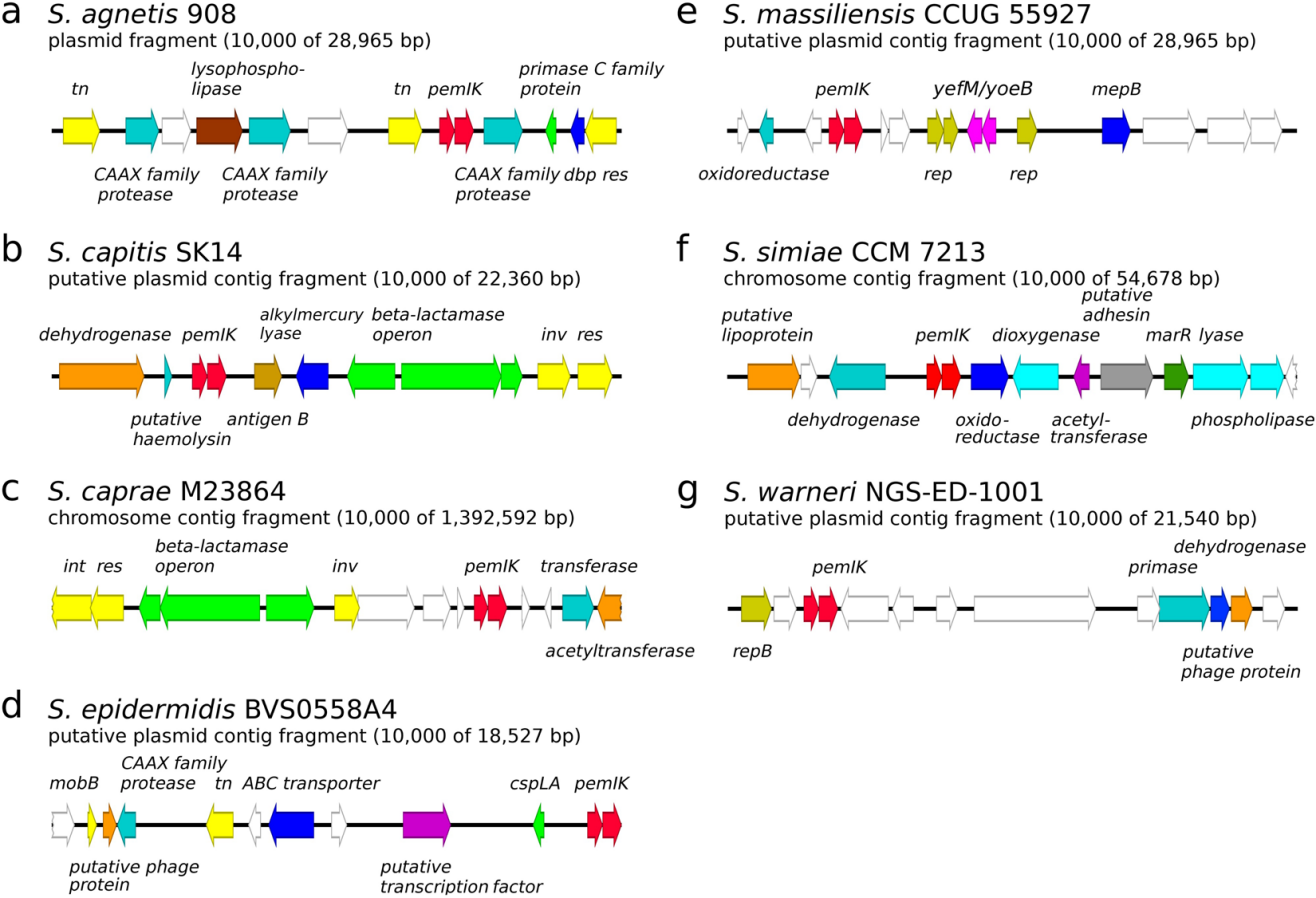
The location of the *pemIK-Sa1* operon within single-strain contexts. White arrows indicate sequences that encode hypothetical proteins, and coloured arrows indicate genes encoding: (**a**) transposase, *tn*, yellow; CAAX family protease, turquoise; lysophospholipase, brown; primase C family protein, green; a putative DNA-binding protein, *dbp*, blue; and resolvase, *res*, yellow. (**b**) Malate dehydrogenase, orange; putative haemolysin, turquoise; putative immunodominant antigen B, light brown; alkylmercury lyase, blue; beta-lactamase resistance, green; invertase, *inv*, and resolvase, *res*, yellow. (**c**) Invertase, *inv*; and resolvase, *res*, yellow; beta-lactamase resistance, green; GNAT family acetyltransferase, turquoise; and 1,4-dihydroxy-2-naphthoate octaprenyltransferase, orange. (**d**) Protein involved in plasmid mobilisation, *mobB*, yellow; putative phage protein, orange; CAAX family protease, turquoise; transposase, *tn*, yellow; teichoic acid ABC transporter permease, blue; putative transcription factor, purple; and cold shock-like protein, *cspLA*, green. (**e**) NADH:ubiquinone oxidoreductase, turquoise; replication proteins, *rep*, olive; *yefM* */yoeB* toxin-antitoxin system, magenta; and MepB protein, *mepB*, blue. (**f**) Putative lipoprotein, orange; D-lactate dehydrogenase, turquoise; NAD(P)H:flavin oxidoreductase, blue; putative ring-cleavage extradiol dioxygenase, sky blue; putative acetyltransferase, purple; putative adhesin, grey; MarR family transcription factor, dark green; putative glyoxylase and carboxylesterase, sky blue. (**g**) Replication protein, *repB*, olive; primase, turquoise; putative phage protein, blue; and aspartate-semialdehyde dehydrogenase, orange.

**Figure 4:**
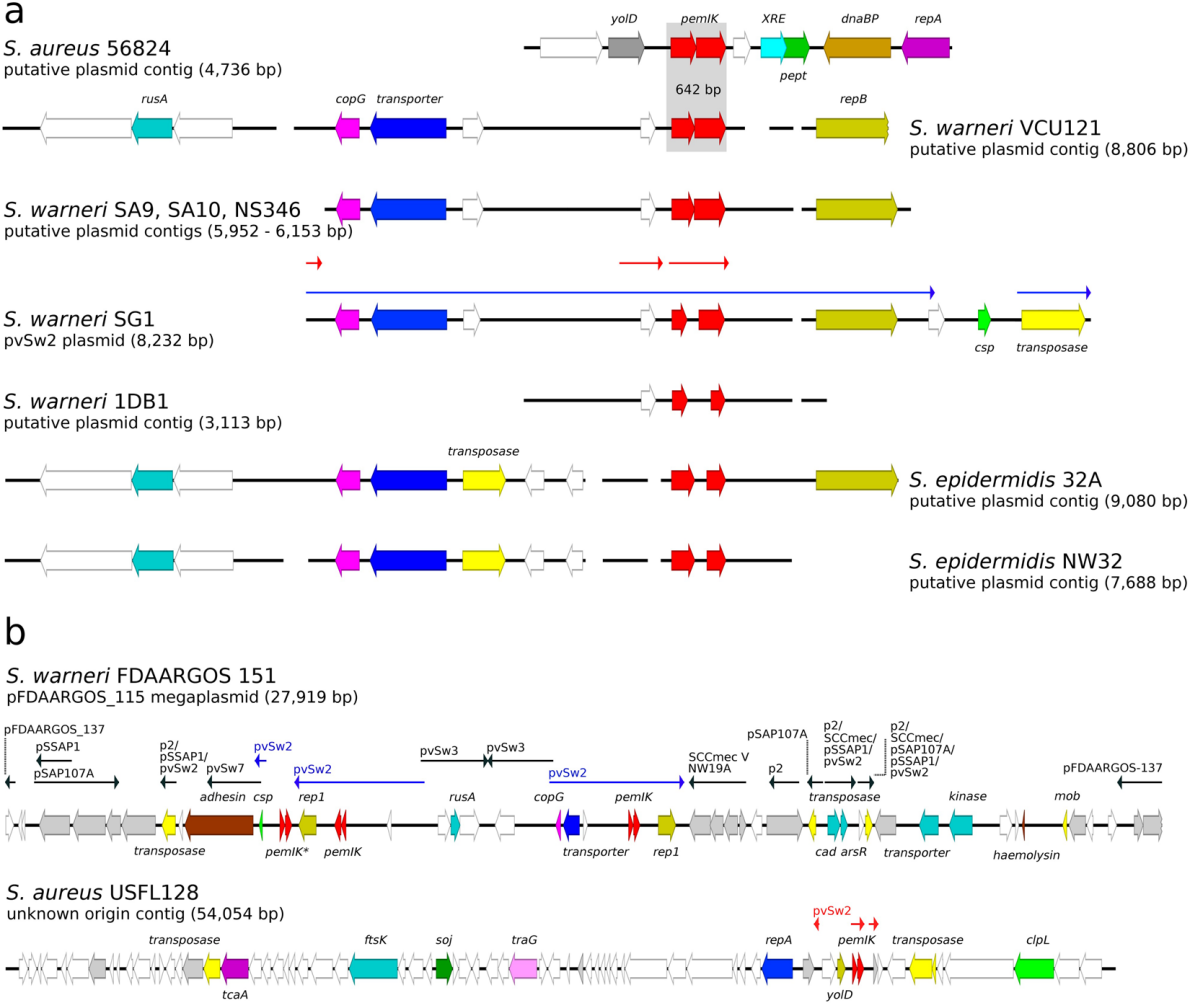
The location of the *pemIK-Swar* operon in *S. aureus*, *S. epidermidis* and *S. warneri* and its proximity to transposase genes. (**a**) The operon is located in the complete pvSw2 plasmid sequence (*S. warneri* SG1) or contigs with high sequence homology to pvSw2 (*S. warneri* SA9, SA10, NS346, 1DBD and *S. epidermidis* 32 A and NW32) as well as in a putative plasmid fragment that was derived from *S. aureus* 5824. Thin red arrows indicate sequence homology to the plasmid pFDAARGOS 151 (panel b). White arrows indicate sequences that encode hypothetical proteins, and coloured arrows indicate the following genes in *S. aureus* 56824: YolD-like protein, *yolD*, grey; *pemIK* locus, red; XRE family transcription factor, cyan; putative peptidase, green; putative DNA-binding protein, *dbp*, light brown; and replication protein A, *repA*, purple. In *S. warneri*, the following sequences are indicated: crossover junction endodeoxyribonuclease RusA, *rusA*, sky blue; CopG family transcription regulation factor, *copG*, magenta; putative peptide transporter, blue; replication protein B, *repB*, olive; cold-shock protein, *csp*, light green; and transposase, yellow. (**b**) The *pemIK-Swar* operon was found as two identical copies in a composite plasmid of *S. warneri* FDAARGOS 151 with the *pemIK-Sa1* operon. Thin arrows indicate sequence homology to other existing plasmids, including pvSw2 (blue, panel a). The operon is also present in a long contig that was derived from *S. aureus* USFL128 that represents a putative plasmid sequence. Thin red arrows indicate sequence homology to pvSw2 (panel a). White arrows indicate sequences that encode hypothetical proteins (light grey) and proteins with different metabolic functions. The selected coloured genes include the following in *S. warneri* FDAARGOS 151: transposase, yellow; adhesin, putative sialoprotein, dark brown; cold-shock protein, *csp*, light green; *pemIK-Sa1* locus, pemIK*, red; replication protein B, *repB*, olive; *pemIK*-*Swar* locus, *pemIK*, red; *rusA*, sky blue; *copG*, magenta; putative peptide transporter, blue; operon involved in cadium transport, *cad*, *arsR*, sky blue; cation transporter and histidine kinase, sky blue; haemolysin, dark brown; and mobilisation protein, *mob*, yellow. In *S. aureus* USFL128: transposase, yellow; teicoplanin resistance-related protein, *tccA*, purple; putative DNA translocase, *ftsK*, sky blue; sporulation initiation inhibitor, *soj*, green; TraG protein, *traG*, pink; *repA*, blue; *yolD*, olive; *pemIK*, *pemIK*-*Swar* locus, red; transposase fragments, yellow; and putative ATP-dependent protease subunit, *clpL*, light green.

The distribution of different TA homologues among species and strains of staphylococci was non-uniform (Tables 1 and 2). Chromosomally encoded *mazEF-Sa* was present in virtually every analysed staphylococcal genome (99.82% of the tested strains) and preceded the operon that encoded the alternative RNA polymerase σ^B^ subunit. We believe that the lack of *mazEF-Sa* in the remaining 11 of 6,132 tested strains is most likely the result of incompleteness in shotgun sequencing data. *pemIK-Sa1* was also characterised by a broad species distribution (positive in 12 out of 36 species tested), but its penetrance was relatively low (only 1.06% of all tested strains). Its low penetrance may be associated with the plasmid-encoded character of *pemIK-Sa1*. However, in certain species (e.g., *S. delphini*, *S. intermedius* and *S. pseudintermedius*), these loci were localised in the chromosome. The 65 identified *pemIK-Sa1* loci that were present in the examined genetic material could be divided into 4 general groups (representing 57 genomes in total; Fig. 2). In the remaining 8 strains, the context was different in each strain (Figs 3 and 4b). Operons related to beta-lactam, arsenic or mercury resistance were identified in the close vicinity of many *pemIK-Sa1* loci (Figs 2 and 3b,c), but the significance of this finding remains unknown. Additionally, the close neighbourhood of many of the *pemIK-Sa1* loci contained genes that encode factors known to be involved in DNA mobilisation, such as DNA invertases, resolvases and transposases (Figs 2a,b,d and 3a–d), suggesting that these elements are potentially mobile. In plasmids (and contigs of likely plasmid origin), the *pemIK-Sa1* loci often coexisted with *yefM/yoeB* loci (Figs 2a,d and 3e), which also belong to class II TA systems. However, the activity of the *yefM/yoeB* loci involves ribosome-dependent endoribonuclease toxins^27^. In one instance, two *pemIK-Sa1* loci neighboured a *pemIK-Swar* locus (Fig. 4b).

**Table 1:** Summary of *mazEF*/*pemIK* loci occurrence among the analysed species and strains.

No.	*mazEF/pemIK* homologue	Number of species	Percent of species	Number of strains	Percent of strains	Location in the analysed strains
1.	*mazEF-Sa*	36	100.00	6121	99.82	chromosome
2.	*pemIK-Sa1*	12	33.33	65	1.06	chromosome, plasmid
3.	*pemIK-Sa2*	1	2.78	8	0.13	undetermined
4.	*pemIK-Sa3*	1	2.78	1	0.02	undetermined
5.	*pemIK-Sa4*	1	2.78	3	0.05	plasmid (putatively)
6.	*pemIK-Sa5*	1	2.78	3	0.05	plasmid (putatively)
7.	*pemIK-Sa6*	1	2.78	1	0.02	chromosome
8.	*pemIK-Scap*	2	5.56	3	0.05	plasmid
9.	*pemIK-Scar*	3	8.33	6	0.10	chromosome (putatively)
10.	*pemIK-Smic*	1	2.78	1	0.02	chromosome (putatively)
11.	*pemIK-Ssci*	2	5.56	7	0.11	plasmid (putatively)
12.	*pemIK-Swar*	3	8.33	11	0.18	plasmid (putatively)
	Total TA carriers:	36	100.00	6121	99.82	
	Total analysed:	36		6132

**Table 2:** Brief summary of the distribution of *mazEF*/*pemIK* loci among the analysed strains.

No.	Species	Number of strains	Average number of *mazEF*/*pemIK* loci	Occurrence of *mazEF*/*pemIK* loci											
				*mazEF-Sa*	*pemIK-Sa1*	*pemIK-Sa2*	*pemIK-Sa3*	*pemIK-Sa4*	*pemIK-Sa5*	*pemIK-Sa6*	*pemIK-Scap*	*pemIK-Scar*	*pemIK-Smic*	*pemIK-Ssci*	*pemIK-Swar*
1.	*S. agnetis*	2	1.50	■	■	□	□	□	□	□	□	□	□	□	□
2.	*S. argenteus*	6	1.00	■	□	□	□	□	□	□	□	□	□	□	□
3.	*S. arlettae*	1	1.00	■	□	□	□	□	□	□	□	□	□	□	□
4.	*S. aureus*	5540	1.01	■	■	■	■	■	■	■	□	□	□	□	■
5.	*S. auricularis*	1	1.00	■	□	□	□	□	□	□	□	□	□	□	□
6.	*S. capitis*	13	1.31	■	■	□	□	□	□	□	■	□	□	□	□
7.	*S. caprae*	2	2.00	■	■	□	□	□	□	□	□	□	□	□	□
8.	*S. carnosus*	3	2.00	■	□	□	□	□	□	□	□	■	□	□	□
9.	*S. chromogenes*	1	1.00	■	□	□	□	□	□	□	□	□	□	□	□
10.	*S. cohnii*	4	1.00	■	□	□	□	□	□	□	□	□	□	□	□
11.	*S. condimenti*	1	2.00	■	□	□	□	□	□	□	□	■	□	□	□
12.	*S. delphini*	1	2.00	■	■	□	□	□	□	□	□	□	□	□	□
13.	*S. epidermidis*	303	1.11	■	■	□	□	□	□	□	■	□	□	□	■
14.	*S. equorum*	4	1.00	■	□	□	□	□	□	□	□	□	□	□	□
15.	*S. gallinarum*	1	1.00	■	□	□	□	□	□	□	□	□	□	□	□
16.	*S. haemolyticus*	172	1.01	■	■	□	□	□	□	□	□	□	□	□	□
17.	*S. hominis*	8	0.88	■	□	□	□	□	□	□	□	□	□	□	□
18.	*S. hyicus*	1	1.00	■	□	□	□	□	□	□	□	□	□	□	□
19.	*S. intermedius*	1	3.00	■	■	□	□	□	□	□	□	□	□	■	□
20.	*S. lentus*	1	1.00	■	□	□	□	□	□	□	□	□	□	□	□
21.	*S. lugdunensis*	7	1.00	■	□	□	□	□	□	□	□	□	□	□	□
22.	*S. massiliensis*	2	1.50	■	■	□	□	□	□	□	□	□	□	□	□
23.	*S. microti*	1	2.00	■	□	□	□	□	□	□	□	□	■	□	□
24.	*S. pasteuri*	3	0.67	■	□	□	□	□	□	□	□	□	□	□	□
25.	*S. pettenkoferi*	1	1.00	■	□	□	□	□	□	□	□	□	□	□	□
26.	*S. pseudintermedius*	4	1.25	■	■	□	□	□	□	□	□	□	□	□	□
27.	*S. saprophyticus*	5	1.00	■	□	□	□	□	□	□	□	□	□	□	□
28.	*S. schleiferi*	4	1.00	■	□	□	□	□	□	□	□	□	□	□	□
29.	*S. schweitzeri*	3	1.00	■	□	□	□	□	□	□	□	□	□	□	□
30.	*S. sciuri*	9	2.33	■	□	□	□	□	□	□	□	□	□	■	□
31.	*S. simiae*	1	2.00	■	■	□	□	□	□	□	□	□	□	□	□
32.	*S. simulans*	2	2.00	■	□	□	□	□	□	□	□	■	□	□	□
33.	*S. succinus*	1	1.00	■	□	□	□	□	□	□	□	□	□	□	□
34.	*S. vitulinus*	1	1.00	■	□	□	□	□	□	□	□	□	□	□	□
35.	*S. warneri*	16	1.69	■	■	□	□	□	□	□	□	□	□	□	■
36.	*S. xylosus*	6	0.83	■	□	□	□	□	□	□	□	□	□	□	□
		Total: 6132	Average: 1.36

**Figure 5:**
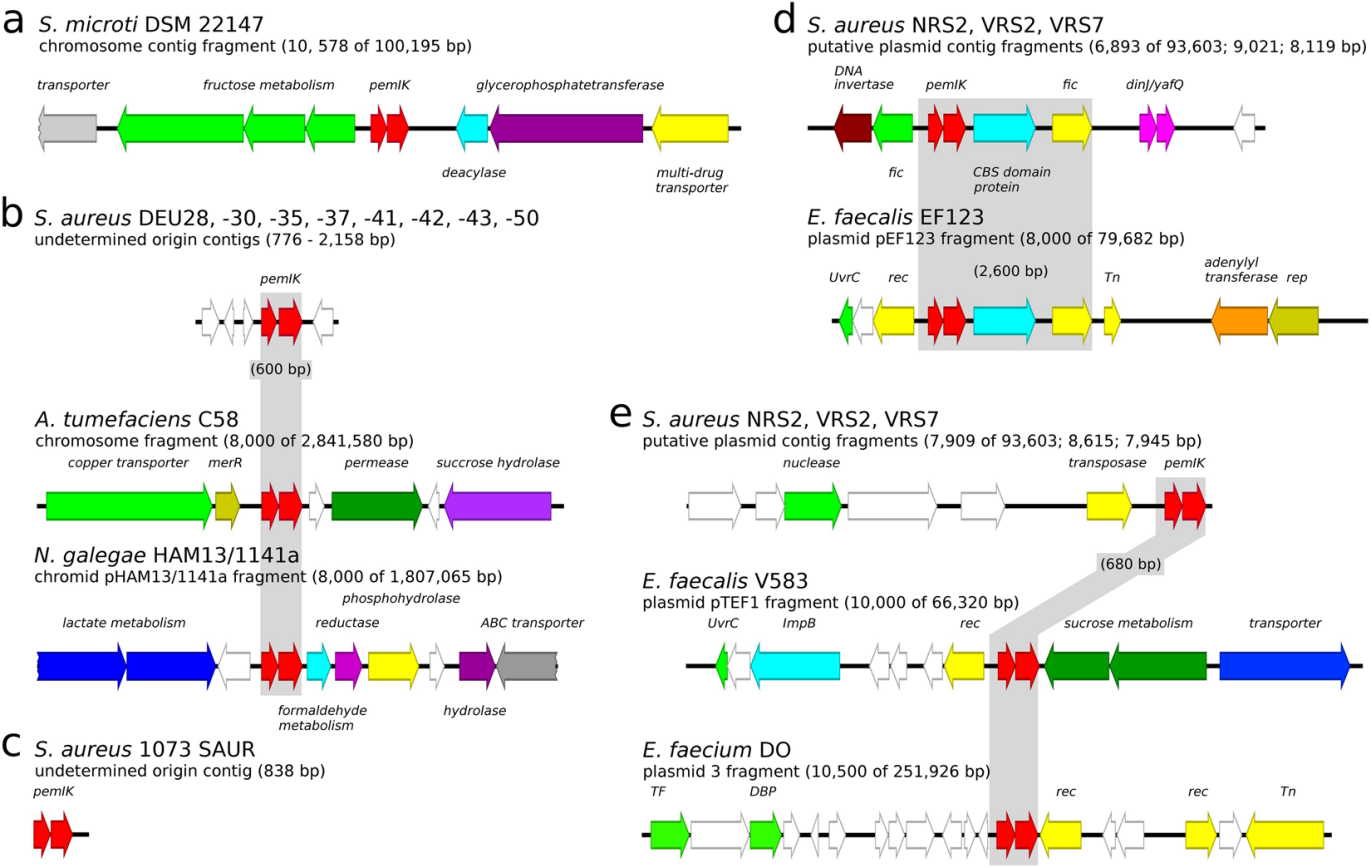
Location of one-species *pemIK* loci. White arrows indicate sequences that encode hypothetical proteins, and coloured arrows indicate the indicated genes. (**a**) A 3′ terminal fragment of a large, likely chromosomal, contig of *S. microti* DSM 22147 that contained the *pemIK-Smic* operon in the context of the following genes: transporter-associated domain protein, grey; operon involved in fructose transport and metabolism, green; *pemIK* locus, red; cysteinyl-tRNA(Pro) deacylase, cyan; CDP-glycerol:glycerophosphate glycerophosphotransferase, dark purple; and multidrug MFS transporter, yellow. (**b**) A common fragment that spanned the largest portion of short contigs of unknown context contained the *pemIK-Sa2* operon, which was derived from 8 different strains of *S. aureus*. (**c**) A short contig containing the *pemIK-Sa3* operon that was derived from *S. aureus* 1073 SAUR. (**d**) A fragment of the contigs that contained the *pemIK-Sa4* operon in three *S. aureus* strains in close proximity to the *parE* family TA system operon as a part of likely plasmid contig. White arrows indicate sequences that encode hypothetical proteins, and coloured arrows indicate the following genes: DNA invertase pin, dark brown; cell division protein Fic, green; *pemIK* locus, red; two CBS domain protein, cyan; cell division protein Fic, yellow; and the *dinJ*/*yafQ* family TA system. (**e**) The same three *S. aureus* strains contained the *pemIK-Sa5* operon. In the case of the NRS2 strain, the operon is located in the same, putatively megaplasmid, contig as *pemIK-Sa4*, but not in close proximity. White arrows indicate sequences that encode hypothetical proteins, and coloured arrows indicate the following genes: DNA-entry nuclease, green; transposase, yellow; and *pemIK* locus, red.

All other identified loci were characterised by restricted distribution and/or penetrance. A set of *mazEF/pemIK* loci that was characteristic of a single species was identified (Fig. 5). The *pemIK-Smic* locus is encoded in the *S. microti* chromosome near a multi-drug transporter (Fig. 5a). *pemIK-Sa*2 – *Sa5* loci were characteristic of *S. aureus* (Fig. 5b–e) and were characterised by very low penetrance. Even though a large number of *S. aureus* genomic sequences are available, these loci were identified in only one or a few strains, implying that *pemIK-Sa2 – Sa5* may later be discovered in other species, provided that sufficient effort is made to expand sequencing in these species.

The low prevalence *mazEF/pemIK* homologues are interesting in terms of the evolution of these genetic elements. *pemIK-Sa2* is an example of a horizontal transfer among unrelated bacterial species. This TA system is relatively common in the mobile genetic elements of *Agrobacterium*, *Neorhizobium* and *Rhizobium* species (Fig. 5b). Close homologues (70–80% sequence similarity) have been found in *Burkholderia*, *Paraburkholderia* and *Pseudomonas*, but none have been identified in species that are more closely related to staphylococci, either evolutionarily or according to an ecological niche. This finding demonstrates that TA systems may switch hosts in a highly unrestricted manner. Even more interesting, *pemIK-Sa3* was found in only a single *Staphylococcus* strain (Fig. 5c). This operon was relatively widespread in the plasmids of *E. coli*, *K. pneumonie*, *S. dysenteriae* and *S. enterolytica* but was never previously identified in a Gram-positive bacterium. Whether this reflects a relatively recent interspecies jump or is simply an effect of contamination of sequencing sample remains to be determined.

*pemIK-Sa4* and *Sa5* represent horizontal transfer from enterococci. Both occurred together in the putative plasmid contigs of the three following strains: NRS2 and two vancomycin-resistant *Staphylococcus aureus* (VRSA) strains, VRS2 and VRS7. The *pemIK-Sa4* ancestor was found in the pEF123 plasmid of *Enterococcus faecalis* EF123, whereas the *pemIK-Sa5* loci were present in the pTEF1 plasmid of *E. faecalis* V583 and plasmid 3 of *E. faecium* DO. Interestingly, the genomes of NRS2, VRS2 and VRS7 contained other putative plasmid contigs that likely originated from the pLG2 and pWZ909 plasmids of *E. faecalis*, and pJEG040 of *E. faecium*. Even more compelling, the two latter plasmids carry determinants of vancomycin resistance and therefore merit a more extended analysis of their history of transfer and evolution. In terms of MazF/PemK toxin evolution, the PemK-Sa4 and PemK-Sa5 sequences are not closely related (Fig. 1). It is interesting that similar to a number of *pemIK-Sa1* loci, the *pemIK-Sa4* loci are located in close proximity to loci of another TA system, *dinJ/yafQ*, whereas *pemK-Sa5* are found in the direct vicinity of a transposase gene (Fig. 5d,e).

**Figure 6:**
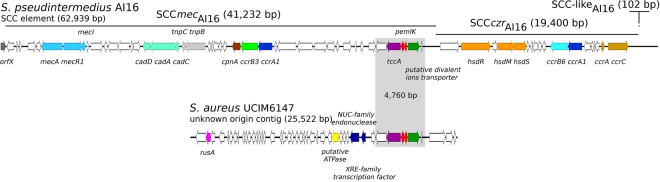
The location of the *pemIK-Sa6* operon in recently described composite staphylococcal cassette chromosome SCC*mec*_AI16_-SCC*czr*_AI16_-CI of *S. pseudintermedius* AI16 and a contig of unknown context that was derived from *S. aureus* UCIM6147. The genes within the composite SSC are illustrated after Chanchaithong *et al*., excluding the *pemIK* locus and two adjacent open reading frames, which were considered hypothetical proteins^29^. White arrows indicate sequences that encode hypothetical proteins, and coloured arrows indicate the following genes: *orfX*, grey; *mec* operon, sky blue; cadium resistance, pale green; cyclopentanol dehydrogenase, brick red; *ccrB3* and *ccrA1* recombinase genes, green and blue; transposases of Tn554 family, light grey; type I restriction-modification system, orange; *czr* operon, brown; *ccrA1*, *ccrB3*, and *ccrB6* recombinase as blue, green, and cyan, respectively. In the sequence of *S. aureus* UCIM6147: crossover junction endodeoxyribonuclease, *rusA*, magenta; putative ATPase, yellow; XRE family transcriptional regulator and a gene located downstream of putative non-specific DNA/RNA NUC endonuclease, dark blue. In the fragment containing the *pemIK* locus: *tccA*, teicoplanin resistance-related protein, dark purple; *pemIK* operon, red; and putative divalent ions transporter, dark green.

Although it was identified in only one *S. aureus* genome, *pemIK-Sa6* is exceptionally interesting. Here, the TA locus was found in the staphylococcal cassette chromosome SCCmec_Al16_, which was previously described in *S. pseudintermedius* AI16^29^ and is in close vicinity to a teicoplanin resistance-related gene (Fig. 6). SCCmecs are mobile genetic elements that have been associated with multi-drug resistance in methicillin-resistant *Staphylococcus aureus* (MRSA)^30^, but their association with TA systems has not been previously reported. PemK-Sa6 is so distantly related to other analysed toxins that its toxin domain has not been detected in *rpsblast*, which relies on direct domain identification within a protein sequence. The operon was, however, identified by all other approaches we used, demonstrating the power of parallel analysis. Because PemK-Sa6 does not contain a PemK domain that was detectable using domain homology searches, these loci were originally annotated as two unrelated hypothetical proteins in the genome of *S. aureus* UCIM147 and in SCC*mec*_AI16_ of *S. pseudintermedius* AI16, demonstrating the utility of focused studies similar to this one. Although the significance of the close genetic linkage observed between *pemIK-Sa4*, *Sa5* and *Sa6* loci and antibiotic resistance elements in MRSA/VRSA remains unclear, these findings clearly merit further investigation.

**Figure 7:**
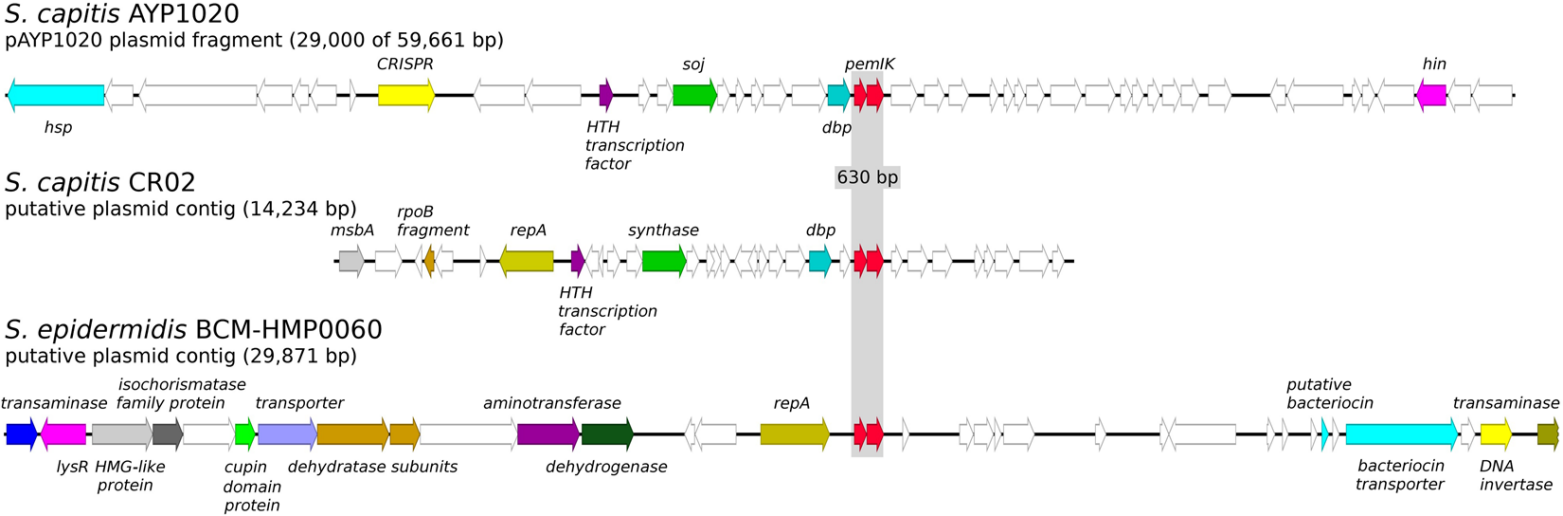
The location of the *pemIK-Scap* operon in *S. capitis* and *S. epidermidis*. The operon was found in three completely different contexts in either a plasmid sequence of pAYP1020 or a putative plasmid sequence. White arrows indicate sequences that encode hypothetical proteins, and coloured arrows indicate the following genes in *S. capitis* AYP1020: heat shock protein 1, *hsp*, cyan; CRIPSR-related DNA-binding protein, yellow; putative HTH transcription factor, purple; sporulation initiation inhibitor protein, *soj*, green; putative DNA-binding protein, sky blue; *pemIK* locus, red; and DNA invertase, hin, magenta. In *S. capitis* CR02: lipid A export ATP-binding/permease protein MsbA fragment, *msbA*, light grey; *rpoB* fragment, beige; replication protein A, *repA*, olive; putative HTH transcription factor, purple; cobyrinic acid a,c-diamide synthase, green; putative DNA-binding protein, sky blue; and *pemIK* locus, red. In *S. epidermidis* BCM-HMP0060: histidinol-phosphate transaminase family protein, blue; LysR family transcriptional regulator, magenta; HMG-like protein, light grey; isochorismatase family protein, dark grey; cupin domain protein, light green; putative transporter, pale purple; *leuC*, *leuD*, 3-isopropylmalate dehydratase subunits, beige; class I/II aminotransferase, dark purple; type I glyceraldehyde-3-phosphate dehydrogenase, *gap*, dark green; replication protein A, *repA*, olive; *pemIK* locus, red; putative bacteriocin and bacteriocin transporter, cyan; putative transposon DNA-invertase Bin3, yellow; and putative histidinol-phosphate transaminase, dark olive.

**Figure 8:**
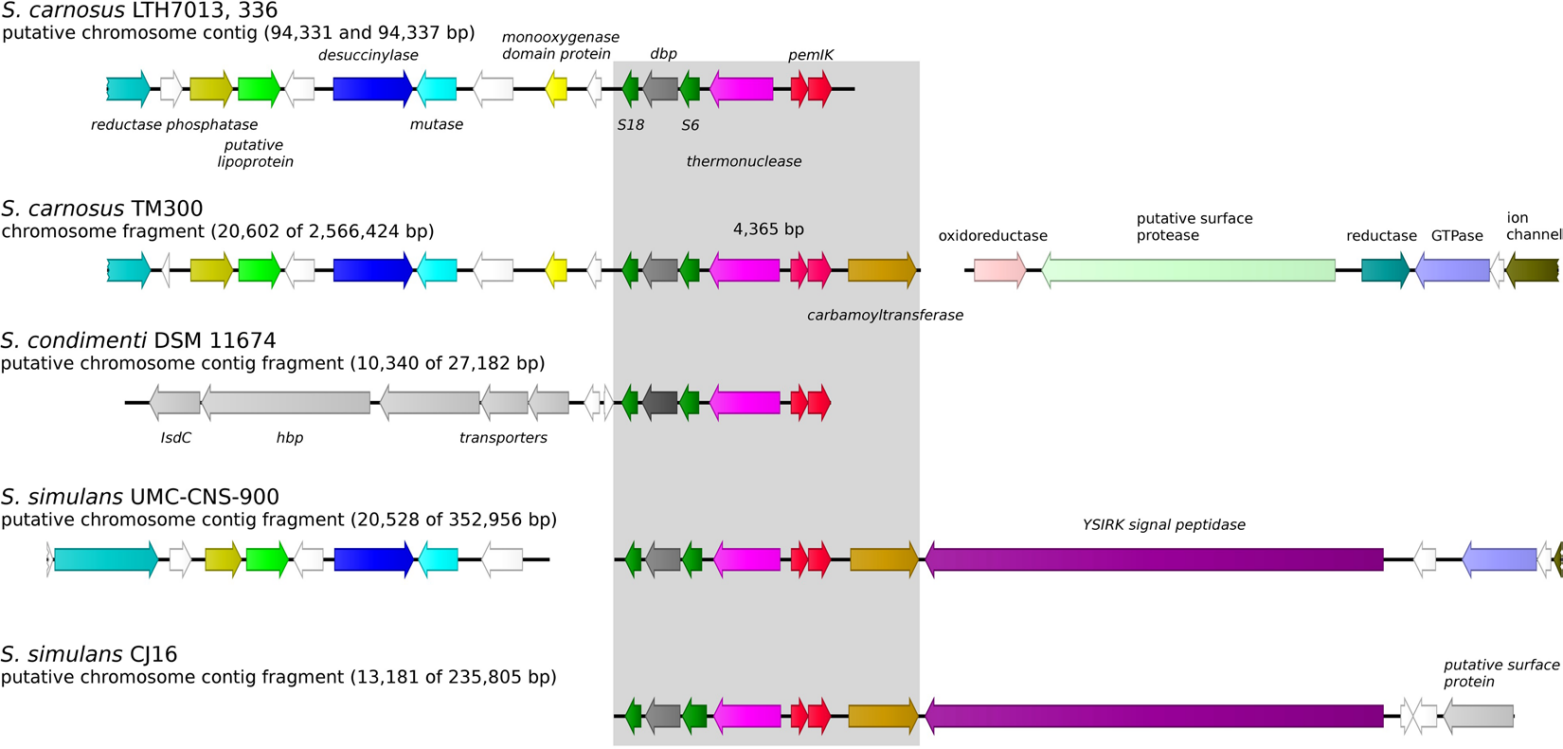
The location of the *pemIK-Scar* operon in *S. carnosus*, *S. condimenti* and *S. simulans*. The cassette containing the *pemIK* operon in the chromosomal sequence of *S. carnosus* TM300 or putative chromosomal contigs. White arrows indicate sequences that encode hypothetical proteins, and coloured arrows indicate the following genes: alkyl hydroperoxide reductase large subunit, *ahpF*, sky blue; putative phosphatase, olive; putative lipoprotein, light green; acetylornithine deacetylase/succinyl-diaminopimelate desuccinylase or related deacylase, blue; fructose-2,6-bisphosphatase, mutase, sky blue; putative protein with monooxygenase domain, yellow; S18 ribosomal protein, green; putative DNA-binding protein, *dbp*, dark grey; S6 ribosomal protein, green; thermonuclease, magenta; *pemIK* locus, red; putative ornithine carbamoyltransferase, *argF*, beige; putative oxidoreductase, pale red; putative surface protease; pale green; putative 5-amino-6-(5-phosphoribosylamino)uracil reductase, *ribD*, sea blue; putative translation factor of GTPase activity, pale purple; putative mechano-sensitive ion channel, ion channel, *mscS*, dark brown; transporters, genes involved in haem metabolism, *isdC*, *hbp*, light grey; YSIRK signal peptidase, purple; and a putative LPXTG-anchored surface protein, light grey.

**Figure 9:**
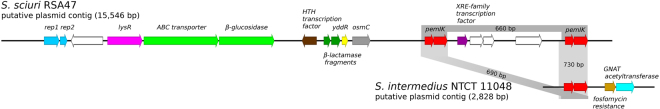
The location of the *pemIK-Ssci* operon in contigs of a putative plasmid sequence of *S. sciuri* RSA47 and a short sequence of *S. intermedius* NTCT 11048 of unknown context. White arrows indicate sequences that encode hypothetical proteins, and coloured arrows indicate the following genes in *S. sciuri* RSA47: fragments of replication protein located in a region homologous to multiple plasmids, *rep1* and *rep2*, sky blue; *lysR* transcription regulator, magenta; β-glucoside ABC transporter components, light green; helix-turn-helix (HTH) transcription factor, dark brown; β-lactamase fragments, dark green; putative transporter permease, *yddR*, yellow; putative peroxiredoxin, *osmC*, grey; *pemIK* locus, red; and XRE-family transcription factor, dark purple. In *S. intermedius* NTCT 11048: *pemIK* locus, red; putative fosfomycin resistance protein, light brown; GNAT-family acetyltransferse, and a putative determinant of aminoglicosides resistance, cyan. In *S. sciuri*, the *pemIK-Ssci* operon is duplicated, but the sequences of the two loci are not identical.

*pemIK-Smic* was found only in *S. microti* and is described above in more detail. The other identified loci were present in more species and were named after the most prevalent one. *pemIK-Scap* was identified in two *S. capitis* strains and one strain of *S. epidermidis* in a plasmid and two likely plasmid contigs, respectively. These loci were not characterised by a common genetic context (Fig. 7), suggesting that they are likely mobile. *pemIK-Scar* was identified in the chromosomes of *S. carnosus*, *S. condimenti* and *S. simulans*. Unlike *pemIK-Scap*, *pemIK-Scar* was present in all of the species within a common genetic context. This conserved cassette exceeds ~4 kbp and contains, in addition to *pemIK*-Scar, two ribosomal protein (S18 and S6)-encoding genes, a putative DNA-binding protein gene, thermonuclease and carbamoyltransferase genes (Fig. 8). *pemIK-Ssci* was identified in several *S. sciuri* strains in likely plasmid contigs. Interestingly, two such loci were present in a single contig. Additionally, *pemIK-Ssci* was identified in a single contig in a *S. intermedius* strain in which it neighboured a fosfomycin resistance gene (Fig. 9). Finally, *pemIK-Swar* was identified in several *S. warneri* plasmids and likely plasmid-derived sequences and a single plasmid sequence that was derived from *S*. *aureus* (Fig. 4a). Additionally, three of these loci were located in putative megaplasmids. One such megaplasmid that was identified in *S. warnerii* contained two *pemIK-Swar* loci and was also neighboured by one *pemIK-Sa1* locus. This instance provides a unique example in which three *pemIK* loci were located in a single plasmid. Another *pemIK-Swar*-containing megaplasmid was identified in *S. aureus* and found to contain only a single TA locus (Fig. 4b).

### Host specificity of *pemIK-Sa1*

We next sought to determine whether any detectable species preference could be associated with any particular TA system. The *mazEF-Sa* loci were present in all examined strains. Loci other than *pemIK-Sa1* were excluded from analysis because of their low penetrance, which precluded a meaningful statistical analysis. Therefore, the analysis was limited to *pemIK-Sa1*. The operon of this TA system was first described in the pCH91 plasmid of a poultry-associated strain^12^. Here, we show that the loci have a clear preference for strains that originate from animals (i.e. non-human origin strains). The reference distribution of all strains across all carrier hosts was significantly different from the distribution of *pemIK-Sa1*-carrying strains (*χ* ^*2*^ test *p* = 0.5, Suppl. Fig. 1). Among the analysed *pemIK-Sa1-*positive strains (65 in total), there was a significant host preference for turkey (*Meleagris gallopavo*) and house and steppe mice (*Mus musculus*, *Mus spicilegus*). This correlation was also reflected in yet another analysis. A phylogenetic tree for all tested strains that was constructed using a gapless *rpoB* alignment (Suppl. Fig. 2) contained a distinct branch of closely related strains (46 in total) that were isolated within a short period of time in a single geographical location (Germany) from chickens, cows, humans, and turkeys. The relationships within this group are so close that the strains were indistinguishable in an *rpoB* analysis and by *spa* typing^31^ apart from individual cases. Interestingly, within this uniform group, the *pemIK-Sa1* loci were found only among poultry isolates. This result is significant because the probability of such a chance distribution is only 5%.

### Unique features of *mazEF-Sa*

The 100% penetrance observed for the *mazEF-Sa* loci in staphylococci is exceptional when compared to our results for other *pemIK* loci. A low diversity of MazF-Sa sequences was evident in our analysis of evolutionary distances (Fig. 1) and represents another substantial distinction between the *mazEF-Sa* and other *pemIK* loci, in which the toxins are much more diverse. Interestingly, the results of a phylogenetic tree constructed using a gapless multiple sequence alignment of *mazF-Sa* sequences (Suppl. Fig. 4) showed that these sequences had the highest log-likelihood of and nearly the same topology as the trees based on *rpoB* and *saoC*. Hence, the diversity observed within *mazF-Sa* genes reflects phylogenetic relatedness among species and had a precision comparable to that obtained using *rpoB* (Suppl. Fig. 2) or *saoC* (Suppl. Fig. 3), which are genes with proven utility for tracing phylogenetic relatedness among staphylococci^32,33^. However, the relatedness among the PemK toxin sequences was not similar to the phylogenetic relatedness between strains and species (Fig. 1). Overall, these data indicate that the *mazEF-Sa* was acquired in a distant past and is currently stably co-evolving within staphylococcal genomes, whereas *pemIK* loci were spread by the horizontal transfer and evolved separately in their host genomes.

### *In vitro* evaluation of the distribution of *mazEF/pemIK* loci in a diverse collection of staphylococcal strains

**Figure 10:**
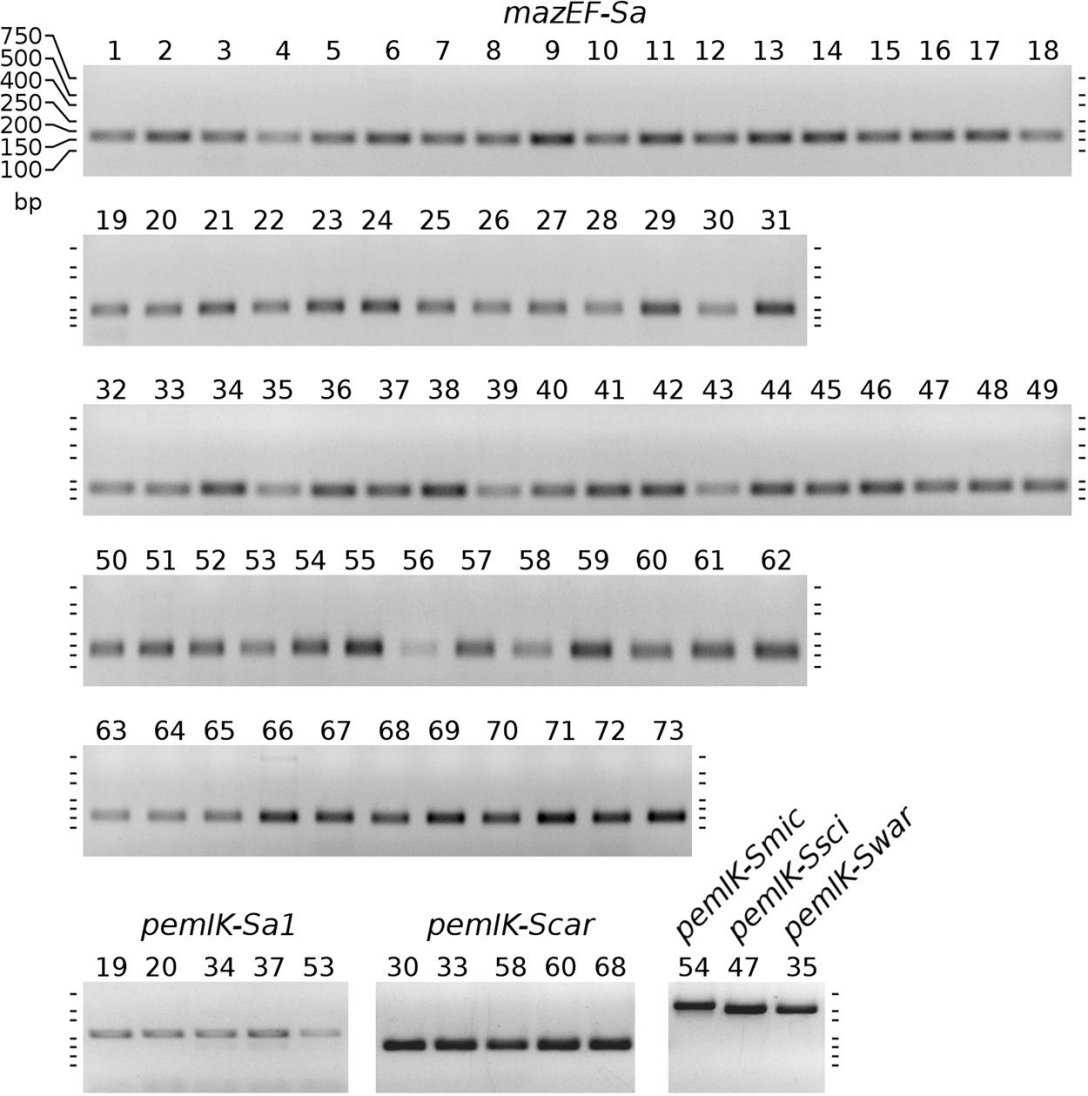
PCR screen for *mazEF/pemIK* loci. The *mazEF-Sa* loci are present in all analysed strains. Other loci are located as follows: *pemIK-Sa1* in *S. aureus* (19, 20)*, S. delphini* (34)*, S. equorum* (37) *and S. massiliensis* (53); *pemIK-Scar* in *S. carnosus* (30)*, S. condimenti* (33)*, S. piscifermentas* (58)*, S. saccharolyticus* (60) *and S. simulans* (68); *pemIK-Smic* in *S. microti* (54); *pemIK-Ssci* in *S. intermedius* (47); and *pemIK-Swar* in *S. epidermidis* (35). The *pemIK-Scap* and *pemIK-Sa2* loci were not found in the analysed strains. The presence of the *pemIK-Sa3* - *Sa6* loci was not verified. The numbers above the lanes correspond to the strain numbers shown in Table 3. For full-length gels see Suppl. Fig. 5.

Although it is most unlikely to occur when using a dataset with a size and diversity similar to that used in this study, one could argue that sequencing or selection bias may have affected the distributions observed in our results. We therefore evaluated our results by experimentally testing the distribution of selected loci in a diverse collection of staphylococcal strains. We designed specific primers based on sequence clustering to detect particular *mazEF/pemIK* loci. PCR was used to determine whether they were present or absent in the genetic material of the tested strains. We carefully designed degenerate primers (Suppl. Table 3) to detect *mazEF-Sa* loci in the tested strains. Because of the high degree of diversity we observed in the sequences, it was not possible to design common primers that would always detect *pemIK-Sa1* loci. Instead, we used a mix of multiple primers that were designed to detect different subgroups, and the results confirmed that a single species possessed *pemIK-Smic* and that multiple species possessed *pemIK-Sa1* and *pemIK-Scar*. In this latter group, the distribution among different spcies was generally supportive of the results of our previous *in silico* analysis. We were also able to detect the presence of *pemIK-Ssci* and *pemIK-Swar* in single strains (Fig. 10; Suppl. Fig. 5).

## Discussion

### Results of homology search approaches

The significant impact of TA systems on bacterial physiology has been extensively investigated over the last three decades. Multiple genome-wide attempts have been made in an attempt to identify and classify new loci in various bacterial species. As a result of the development of massive parallel sequencing techniques, there has been a substantial increase in the number of complete bacterial genomic sequences in the last 5 years. Moreover, the last comprehensive analysis of staphylococcal genomes was performed 8 years ago by Makarova *et al*.^24^; that is, before the era of massive sequencing. Even though the dataset analysed by Makarova and collaborators included all known archaeal and bacterial genomes, only 18 staphylococcal genomes were available at that time, whereas more than 6,000 were available when this study was initiated. The Makarova study identified only a single *mazEF/pemIK* family TA system, *mazEF-Sa*, in staphylococci and concluded that the entire *Bacillales* phylum was not abundant in TA loci^24^. Until recently, only a single additional *mazEF/pemIK* homologue, *pemIK-Sa1*, was described in staphylococci^12^. The recent acquisition of a large number of genomic sequences for staphylococci provided us with a fantastic opportunity to challenge these views and re-evaluate the status of *mazEF/pemIK* TA systems in this genus.

Analysing a large number of biological sequences requires time-efficient analytical tools, such as the heuristic approaches implemented in BLAST family algorithms. BLAST was first introduced in 1990^34^ and has since evolved to include a broad array of tools^35–37^. Approaches based on these tools now dominate database search routines. In this study, we used and compared the results of the most relevant approaches.

Our aim in performing a conservative cascade search using a protein BLAST approach was to identify loci characterised by close relatedness. This search was conducted based on a simple and intuitive parameter involving sequence similarity in which the threshold was arbitrarily set to 50%. Sequence similarity is inherently associated with each pair of sequences and is independent of score matrix and database size, opposed to the E-value (and the mathematically interrelated score value), which is the main statistic of significance provided with BLAST results and is calculated based on random simulations performed since the introduction of gapped BLAST^35^. Protein BLAST is moreover not well tailored for searches for remote homologues^35,38,39^. Only an iterated cascade search, which turns the outcomes of one search into a query for another search, enables the discovery of distant homologues. Our use of this approach resulted in the identification of 10 new *mazEF/pemIK* homologues within the analysed dataset of all available staphylococcal genomes. We considered this group a reference for extensive approaches aimed at discovering less-related sequences that present a risk of false positive results.

**Figure 11:**
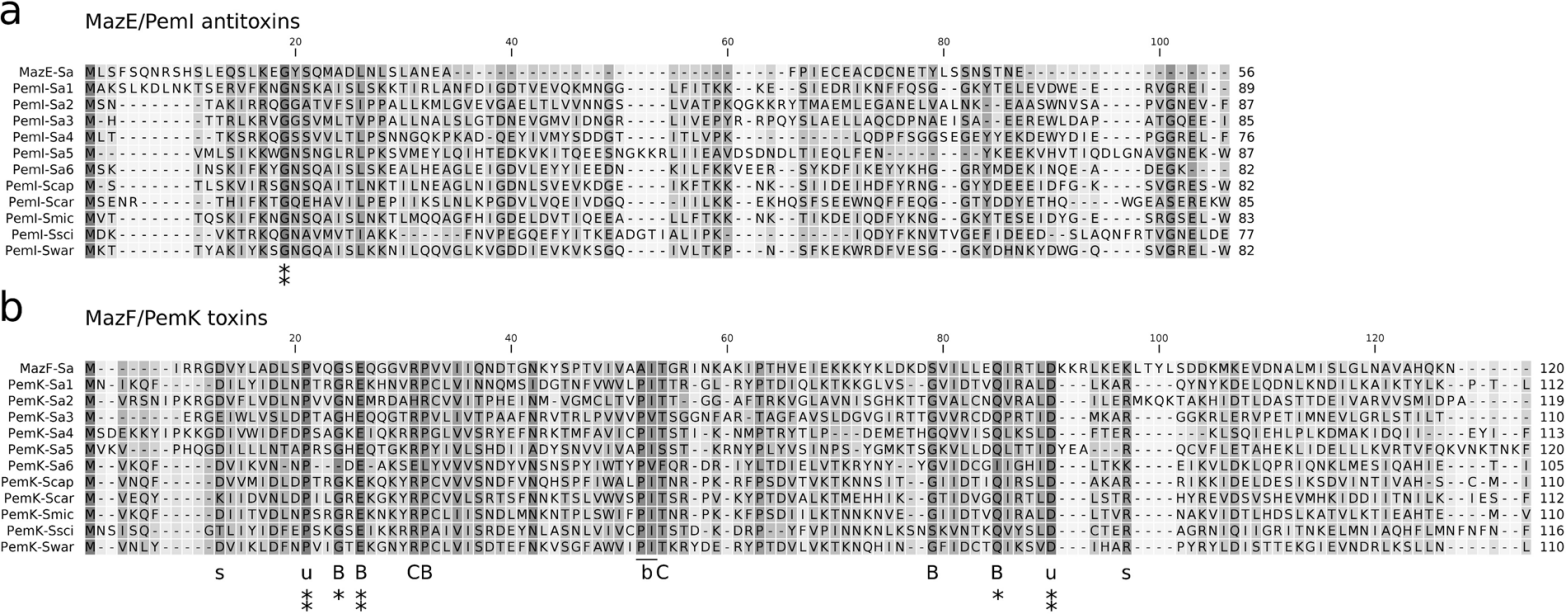
Conservation among (**a**) MazE/PemI and (**b**) MazF/PemK protein sequences. Residues that were highly conserved across all sequences are marked by a double asterisk, whereas those conserved among toxin sequences (excluding PemK-Sa6) are marked by a single asterisk. Letters below the alignment refer to conserved residues previously described as: *B*, residues engaged in substrate binding; *C*, residues suggested as catalytic (based on structures of MazF from *Bacillus subtilis* and *Escherichia coli* ^62,63^) or residues putatively: *b*, engaged in substrate binding; *s*, responsible for structural stability; *u*, solvent exposed residues of unknown role (based on our inspection of available 3D structures of MazF/PemK toxins).

Many approaches incorporated in subsequent analyses have used BLAST tools and less stringent E-value thresholds. The E-value represents the number of hits with a score at least as high as the given one that would be expected purely by a chance^37^. Hence, generously defining the E-value cutoff increases the hit rate but at the expense of false positives (i.e., randomly correlated sequences). In this regard, our approaches resembled those previously used by Sevin *et al*.^21^ and Makarova *et al*.^24^. Obtained results were filtered to identify sequence pairs that encoded potential *mazEF/pemIK* TA systems. Our first approach was to use protein BLAST. The second approach involved PSI-BLAST, which tests for homologous sequences in an iterative fashion. In the first iteration, a simple protein BLAST search is performed, and all of the results that exceed a certain inclusion threshold are aligned and subsequently used to prepare a position-specific score matrix (PSSM). The PSSM serves as a query and is refined after each iteration. This approach allows the incorporation of information related to the substitution frequencies that are intrinsic to a particular family of protein sequences instead of a general substitution matrix, such as BLSUM62, which is the default matrix that is used for protein BLAST. Additional approaches used in this study included RPS-BLAST, which tests a query sequence against a database of PSSMs that have been predefined for various protein families, and DELTA-BLAST, which first queries a PSSM database with a sequence and then uses matched PSSMs to search a protein database. Use of the PSI-BLAST, RPS-BLAST and DELTA-BLAST approaches facilitate the discovery of distant homologues, whereas PSI-BLAST is carefully optimised to limit false positives to the greatest extent possible^35,39,40^. This approach, especially the inclusion of PSI-BLAST-based cascade searches, is why remarkable levels of sensitivity and specificity can be achieved^41^. Creating a PSSM from scratch provides a considerable advantage that was demonstrated during our search for PemK-Sa6 sequences. Even though this sequence shares significant homology with the other staphylococcal PemK toxin sequences described in this study, no NCBI CDD conservative domain was detected within the sequence, likely because of specific amino acid substitutions (Fig. 11). This characteristic resulted in a false negative in the RPS search; indeed, PemK-Sa6 was only found because it is located in close vicinity to PemI-Sa6, an antitoxin with a detectable MazE domain. Moreover, PSI-BLAST readily identified PemK-Sa6 with no additional assumptions regarding its genetic neighbourhood. These findings for PemK-Sa6 demonstrate that although all extensive approaches return a similar general list of results (hence, only a single, arbitrarily chosen one is usually used, as in^21,24,42^), a comprehensive analysis requires the parallel use of all of these approaches to ensure the identification of all rare examples.

### Staphylococci carry at least 12 independent *mazEF/pemIK* homologues

In the end, all of the extensive methods tested in this study yielded results similar to those achieved using a conservative cascade search with protein BLAST. We identified 10 novel *mazEF/pemIK* homologues in addition to the previously known two. These results clearly indicate that in staphylococci, the *mazF/pemK* family forms a distinct and coherent group of relatively closely related members. Interestingly, the protein sequences of antitoxins within different TA families vary substantially and are therefore of limited use in finding homologues and devising a system of classification for TA systems. This proposal is in agreement with the previous findings of Makarova *et al*.^24^ and supported by the findings presented here (Fig. 11).

Our threshold of 80% toxin protein sequence similarity to identify novel *mazEF/pemIK* homologues yielded sets (loci) in which the two most dissimilar sequences were of 95% similarity. This approach is compelling because the results of using this system of classifying *mazEF/pemIK* loci corresponded to their actual genomic context and the similarity threshold was above the similarity levels observed among toxin sequences encoded by distinct homologous loci, which is 78% between the two most similar observed sequences that belonged to different *mazEF/pemIK* homologues.

*mazEF-Sa* is undoubtedly unique because it is widespread throughout all staphylococcal genomes. Moreover, the sequence of the MazF-Sa toxin is far more conserved among different species than the sequences encoded by other *pemIK* loci. Additionally, we found that the phylogenetic relatedness of *mazF-Sa* sequences corresponded to the phylogenetic relatedness of the respective genomes. In fact, the chromosomal locations and strong coupling between the functions of the *mazEF-Sa* operon and the alternative sigma subunit B of RNA polymerase (SigB, σ^B^)^43^ suggest that there has been a long period of coevolution between these sequences. The *mazEF-Sa* operon proceeds the *rsbUVWsigB* operon, and the first is necessary for the full activity of SigB, whereas SigB negatively regulates *mazEF-Sa* transcription^44^. Both operons are co-transcribed^44,45^. Finally, the functions of *mazEF-Sa* that are associated with stress-induced persister cell formation and beta-lactam sensitivity^46^ are highly correlated with the functions of SigB under stress conditions^47^.

### Maintenance of mobile genetic elements and *pemIK* loci

*pemIK* loci were initially recognised as plasmid maintenance systems^6–8^. The loss of a TA-carrying plasmid leads to toxin release by degradation of the unstable antitoxin. A cell lacking a *pemIK* locus is unable to replenish the antitoxin pool, resulting in inhibited growth and eventual cell death. Hence, daughter cells that would not inherit a TA-encoding plasmid are not produced. The DNA maintenance property of TA systems is broader than that of the plasmids alone. Whereas most staphylococcal *pemIK* loci are located in plasmids (including megaplasmids or multiple loci per plasmid), *pemIK* loci have a stabilising effect on the genetic neighbourhood within the same bacterial chromosome^48,49^. A *pemIK-Sa2* locus is present in a chromid (a chromosome-like replicon that may exceed 1 Mbp in size) in *Neorhizobium* ^50^. If *pemIKs* are capable of promoting the maintenance of these large genetic elements, it may also have a significant influence on the maintenance of antibiotic resistance. The *pemIK* loci have been found to be located in close proximity to antibiotic-resistance genes or in mobile genetic elements that carry such genes, especially the SSC*mec* element and probably plasmids that carry vancomycin-resistance genes. The maintenance of these genetic elements and the role played by TA systems in this process is clinically relevant in the context of MRSA and VRSA strains. Of particular interest are *pemIK-Sa4* and *pemIK-Sa5* loci. In *S. aureus*, vancomycin resistance has been proposed to arise as a result of horizontal transfer from vancomycin-resistant enterococci (VRE)^51,52^. The exchange of fragments among plasmids and the aggregation of fragments into megaplasmids appear to be common occurrences, as shown in Fig. 4b for pDFAARGOS_115 of *S. warneri*. In this context, it seems likely that both the *pemIK-Sa4* and *pemIK-Sa5* loci were transferred from enterococci along with vancomycin-resistance genes, and these are likely embedded into the same putative plasmids. Although the *pemIK* loci have been identified next to vancomycin-resistance determinants in different putative plasmid contigs in VRSA strains, the potential functional relationship between these elements requires further investigation. It seems likely that particular *pemIK* TA systems preserve functionality even when transferred to distantly related species. We believe so because the non-coding regions vary significantly more between different species than the coding sequences (Suppl. Fig. 6). The non-coding sequences contain potential promoters which must adapt to particular species while the coding regions remain stable, further suggesting evolutionary pressure to preserve function.

### Regulatory role of *mazEF/pemIK* homologues

In addition to its role in maintaining genetic elements, multiple lines of indirect evidence suggest that PemK toxins play a regulatory role in modulating the bacterial transcriptome and therefore gene expression. This regulation seems particularly relevant during stress-induction in TA systems and during persister formation by pathogenic bacteria^53–57^. This presumed regulatory role is likely to be related to the endoribonucleolytic activity of the PemK toxin. PemKs have been demonstrated to be ribosome-independent mRNA interferases, but the role of this process in the regulation of orchestrated gene expression remains elusive at the experimental level. *S. aureus* MazF-Sa and its homologue form in *S. equorum* show stringent specificity for the pentanucleotide sequence UACAU^10,28^. PemK-Sa1 recognises the tetranucleotide sequence UAUU^12^. MazF homologues in other bacteria recognise sequences between 3 and 7 nucleotides in length^9,10,58–60^. It is unlikely that such stringent specificity evolved in enzymes that are physiologically involved in unspecific degradation of total RNA. It has instead been speculated that many PemK toxins target specific gene pools, whereas other pools evolved that did not contain the target sites. Hence, PemKs could thereby globally regulate gene expression upon TA system activation^9,10,12^. In the results presented, a broad array of staphylococcal *pemIK* loci were identified (Fig. 1).

The protein sequence similarity among different MazF/PemK homologues varies from 22 to 78%. A question arises whether our clustering criteria relate to functional differences? Even though very few well characterized examples are available, the 45% similarity associates with different cleavage specificities of MazF-Sa and PemK-Sa1^10,12^. To the opposite, 85% similarity characterizes two proteins of identical specificity, MazF-Sa and MazF-Bs^10,61^. The above levels correspond to the homology criteria adopted in our work according to which toxins from different strains were classified into a single or different groups. It is thus likely that most, if not all newly uncovered MazF/PemK homologues target different RNA sequences. But are they functional at all? Analysis in the context of structures of MazF-Bs and MazF-Ec^62,63^ demonstrates that the novel homologues preserve some conserved residues, including those responsible for substrate binding and catalysis (Fig. 11) suggesting functional role. These conclusions are however highly speculative and future experimental characterization is clearly necessary.

### Host preference of *pemIK* loci

With the exception of the *pemIK-Sa1* loci, the low frequency of most of the *pemIK* loci in staphylococci did not enable us to determine host preference. It was previously suggested that *pemIK-Sa1* exhibits a preference for non-human hosts, especially poultry^64^. This TA system was first identified in the pCH91 plasmid, which is a homologue of pAvX, a plasmid characteristic of poultry strains of *S. aureus* ^64^. The results of our current investigation demonstrate that *pemIK-Sa1* occurs in a number of different contexts and primarily in plasmids and putative plasmid contigs. However, the host preference of *pemIK-Sa1* is not completely clear from our statistical analysis. This question is sufficiently compelling to be considered in future studies.

## Methods

### Bioinformatic analyses

Staphylococcal genomic sequences, including both complete and shotgun contigs, were retrieved from the GenBank database^65^. The complete list of analysed sequences, including accession numbers, is provided in Suppl. Table 1. Sequences from other species that were used in the accessory analyses are listed in Suppl. Table 2. The computational analysis was performed using self-developed Python/IPython scripts using standalone NCBI BLAST + tools 2.3.0^66^ with default parameter values unless otherwise indicated. Open reading frames (ORFs) were identified and translated using multi-threaded functions developed in C++ and deployed as a Python module. A naïve search for all possible ORFs with a minimal length of 100 bp was performed for all sequences. The alternative start codons ATA, ATC, ATG, ATT, CTG, GTG and TTG were considered in parallel to the canonical ATG. To minimise redundancy, only the longest variant of each ORF was further analysed. The ORFs were translated to protein sequences using a bacterial codon table 11^67^. Translated ORFs were probed for homology to well-characterised toxins and antitoxins in the MazEF/PemIK family, including MazEF-Sa^10^ and PemIK-Sa1^12^. Five different approaches that were based on functionalities provided by BLAST+ tools were used as described below.

### Conservative cascade search using protein BLAST

A BLAST database was created using *makeblastdb* for each set of translated ORFs representing a single bacterial strain. Each database was queried using the protein sequences of MazEF-Sa and PemIK-Sa1 components using the *blastp* tool with the E-value threshold of 0.1. The threshold for homologues was arbitrary set at a sequence similarity greater than 0.80 (corresponding to 0.60 sequence identity) and a match-to-query length ratio between 0.55 and 1.65. Matching sequences that met the length ratio requirement but had similarity below 0.80 but greater than 0.50 were further manually analysed. The presence in a two-gene operon and an arrangement typical of TA systems (i.e., genes partially overlapping or less than 100 bp apart) were initially used as criteria to classify such loci as a hypothetical TA system. Further criteria included the presence of a complete MazF/PemK domain (defined according to NCBI Conserved Domain Database v3.14)^68^ in the putative toxin sequence or its length within 80 to 150 amino acids (aa) or the presence of a complete MazE domain in the putative antitoxin sequence or its length within a range of 50 to 100 aa. Newly discovered TA homologues were used to iterate the procedure until convergence, which was defined as when no new putative *mazEF/pemIK* loci were identified.

### Extensive search using protein BLAST

Preparation for and the initial search of the database were performed using the same method as was used for the conservative cascade method. For each sequence match, protein sequences encoded by adjacent ORFs and arranged in a manner typical of TA systems (ORFs overlapping or closer than 100 bases) were added to the result list. This ensured the inclusion of potential TA system components in instances wherein only a single one was closely matched to the query sequence. The resulting set of protein sequences was clustered using *blastclust* with a length coverage threshold of 0.55 and a sequence identity threshold of 60%. Pairs of clusters containing ORFs that co-localised at any frequency in the analysed genomes were classified as potential TA systems. The final inclusion criteria were identical to those used in the conservative cascade method.

### PSI-BLAST search

The procedure was identical to that used for the extensive search except that *psiblast* was used instead of *blastp*. PSI-BLAST is specifically tailored to identify distant homologues. To achieve this goal, it uses a position-specific matrix that is updated during each step of the iterative search procedure, unlike protocols that use a predefined score matrix (i.e., for a protein BLAST).

### RPS-BLAST search

An RPS database containing the domain profiles that are present in components of *mazEF/pemIK* TA systems (e.g., COG2336, COG2337, pfam04014 and pfam02452) was created using *makeprofiledb*. The database was queried for all translated ORFs using *rpsblast* with the E-value threshold of 0.001. The ORF sets were prepared and subsequent steps were identical to those used to perform the conservative cascade method.

### DELTA-BLAST search

DELTA database was created in a manner comparable to that used for the RPS database. The database search and the processing of results were performed in a manner identical to that used to perform the extensive search method except that *deltablast* was used instead of *blastp*.

### Analysis of the genetic neighbourhood of *mazEF/pemIK* family loci

The genetic context of each identified TA loci was manually analysed using a graphical interface for the BLAST tools and implemented in CLC Main Workbench (CLC Bio/Qiagen). The analysis was based on the results of blast searches, annotation browsing, multiple sequence alignments and phylogenetic tree construction, and this allowed us to define particular genetic contexts. The figures were prepared based on graphics that were created in CLC Main Workbench and further processed using GIMP.

### Construction of phylogenetic trees

The sequences for the MazF/PemK toxins identified in all relevant strains, the *mazF-Sa*, *rpoB* and *saoC* genes in strains carrying the *pemIK-Sa1,* belonging to a group of closely related strains that were isolated in Germany and included in the BioSample database^69,70^, were used to analyse phylogenetic relationships. Toxins determine the biological activity of a particular TA system, and their sequences are less variable among different strains. Furthermore, one antitoxin family may be coupled with just a few toxin families^24^. For these reasons, only toxin sequences were used in the phylogenetic analyses. All unique sequences were aligned in CLC Main Workbench (see supplementary data for detailed alignment parameters). Segments of the alignment containing gaps were removed, as were additional duplicates that arose after gaps were removed. Based on the resulting alignments, phylogenetic trees were constructed using the Maximum Likelihood Phylogeny tool in CLC Main Workbench. The model was chosen using the Model Testing tool (see the supplementary data for the details associated with the tree construction method).

### Host distribution analysis

For all analysed strains, the collection date, geographical location and host name were retrieved from the BioSample database using self-developed Python scripts and the NCBI *esearch* and *efetch* tools^71,72^. BioSample accession numbers were extracted from cross-referenced fields obtained from GenBank records whenever available. In the remaining cases (161 strains), the BioSample database was queried using the organism name and the strain/isolate signature provided within the GenBank record.

### Experimental identification of *mazEF/pemIK* loci

*Staphylococcus* strains were obtained from international reference collections, including ATCC, BCCM/LMG and DSMZ, and the Polish Collection of Microorganisms (PCM, Wroclaw, Poland), as indicated by strain signatures, and from the collections of the Department of Microbiology, Faculty of Biochemistry, Biophysics and Biotechnology, Jagiellonian University^33,73^ (Table 3). The bacteria were cultured overnight in tryptic soy broth (TSB, Sigma-Aldrich) at 37 °C at 180 RPM. To isolate DNA, bacterial pellets obtained from 2 ml cultures were suspended in 200 μl of EC buffer (6 mM Tris-HCl pH 7.6; 1 M NaCl; 100 mM EDTA; 0.5% Brij; and 0.5% sarkosyl) supplemented with 1 μl of RNase A (10 mg/ml, Thermo Scientific) and 1 μl lysostaphin (10 mg/ml, Preparatis) and incubated for 1 h at 37 °C. After the lysis was completed, the DNA was isolated using a Genomic Mini Kit (A&A Biotechnology) according to the manufacturer’s protocol. Fragments of different *mazEF/pemIK* loci were PCR amplified from 50-100 ng genomic DNA using specific primers (1 μM; Suppl. Table 3) and the following PCR cycling parameters: initial denaturation (94 °C, 2 min), cycle-specific denaturation (94 °C, 30 sec), an annealing temperature appropriate for a particular set of primers (Suppl. Table 3) for 30 sec., and extension (74 °C, 1 min). The three latter steps were repeated 29 times and were then followed by a polishing step (74 °C, 10 min). DNA Polymerase (1 U, A&A Biotechnology) and manufacturer-supplied buffers were used. The PCR products were separated on 1% agarose gels in TAE buffer (40 mM Tris, 20 mM acetic acid, and 1 mM EDTA).

**Table 3:** List of strains used in PCR screens.

No.	Species	Strain	No.	Species	Strain
1.	*S. agnetis*	DSM 23656	38.		LMG 19116
2.	*S. argenteus*	DSM 28299	39.	*S. felis*	PCM 2408
3.	*S. arlettae*	LMG 19112	40.	*S. gallinarum*	LMG 19119
4.		LMG 19113	41.		LMG 19120
5.	*S. auricularis*	PCM 2428	42.		PCM 2439
6.	*S. aureus*	2581 (co08)	43.	*S. haemolyticus*	DM-18
7.		2902(do1)	44.	*S. hominis*	ATCC 27844
8.		53 (32)	45.		LMG 26014
9.		85 (59)	46.	*S. hyicus*	PCM 2192
10.		A25698	47.	*S. intermedius*	ATCC 29663
11.		B/23647/363 cat	48.	*S. kloosii*	ATCC 43959
12.		D-6S	49.		LMG 19132
13.		GD12	50.		LMG 19133
14.		GD23	51.	*S. lentus*	PCM 2441
15.		GD27	52.	*S. lugdunensis*	ATCC 43809
16.		M-horse (ho1)	53.	*S. massiliensis*	DSM 23764
17.		Mastitis 11	54.	*S. microti*	DSM 22147
18.		Poitou donkey (dn1)	55.	*S. muscae*	PCM 2406
19.		pa3	56.	*S. pasteuri*	PCM 2445
20.		ph2	57.	*S. pettenkoferi*	DSM 19554
21.		Tapir ZOO	58.	*S. piscifermentas*	ATCC 51136
22.		VA21332/04	59.	*S. pulvereri*	PCM 2443
23.		VA544/05	60.	*S. saccharolyticus*	PCM 2529
24.		VA589/05	61.	*S. saprophyticus*	ATCC 15305
25.		VA727/05	62.		ATCC 49453
26.		VS50	63.	*S. schleiferi*	LMG 19137
27.		VS56	64.		LMG 22205
28.	*S. capitis*	ATCC 27840	65.		PCM 2426
29.	*S. caprae*	ATCC 35538	66.	*S. sciuri*	ATCC 29062
30.	*S. carnosus*	ATCC 51365	67.	*S. simiae*	DSM 17636
31.	*S. chromogenes*	PCM 2193	68.	*S. simulans*	PCM 2106
32.	*S. cohnii subsp. cohnii*	PCM 2108	69.	*S. succinus casei*	DSM 15096
33.	*S. condimenti*	DSM11674	70.	*S. succinus succinus*	DSM 14617
34.	*S. delphini*	ATCC 49172	71.	*S. vitulinus*	PCM 2470
35.	*S. epidermidis*	ATCC 14990	72.	*S. warneri*	ATCC 27836
36.		ATCC 25547	73.	*S. xylosus*	ATCC 29971
37.	*S. equorum*	ATCC 43958			

## Declarations

### Availability of data and materials

All data supporting the conclusions presented in this article are available at the GenBank FTP site (ftp://ftp.ncbi.nlm.nih.gov/genomes/genbank/bacteria/) or the NCBI Nucleotide website (https://www.ncbi.nlm.nih.gov/nucleotide/). The accession numbers of the analysed genomes are summarised in Suppl. Table 1.

## Electronic supplementary material

Supplementary data

Supplementary tables

## References

[1] Bukowski M, Rojowska A, Wladyka B (2011). Prokaryotic toxin-antitoxin systems–the role in bacterial physiology and application in molecular biology. Acta Biochim Pol..

[2] Schuster CF, Bertram R (2013). Toxin-antitoxin systems are ubiquitous and versatile modulators of prokaryotic cell fate. FEMS Microbiol Lett..

[3] Unterholzner SJ, Poppenberger B, Rozhon W (2013). Toxin–antitoxin systems: biology, identification, and application. Mob Genet Elements.

[4] Wen Y, Behiels E, Devreese B (2014). Toxin-antitoxin systems: their role in persistence, biofilm formation, and pathogenicity. Pathog Dis..

[5] Schuster C, Bertram R (2016). Toxin-antitoxin systems of Staphylococcus aureus. Toxins (Basel)..

[6] Gerdes K (1986). Mechanism of postsegregational killing by the hok gene product of the parB system of plasmid R1 and its homology with the relF gene product of the E. coli relB operon. EMBO J..

[7] Tsuchimoto S, Ohtsubo H, Ohtsubo E (1988). Two genes, pemK and pemI, responsible for stable maintenance of resistance plasmid R100. J Bacteriol..

[8] Sobecky PA, Easter CL, Bear PD, Helinski DR (1996). Characterization of the stable maintenance properties of the par region of broad-host-range plasmid RK2. J Bacteriol..

[9] Zhu L (2008). The mRNA interferases, MazF-mt3 and MazF-mt7 from Mycobacterium tuberculosis target unique pentad sequences in single-stranded RNA. Mol Microbiol..

[10] Zhu L (2009). Staphylococcus aureus MazF specifically cleaves a pentad sequence, UACAU, which is unusually abundant in the mRNA for pathogenic adhesive factor SraP. J Bacteriol..

[11] Van Melderen L, Saavedra De, Bast M (2009). Bacterial toxin-antitoxin systems: more than selfish entities?. PLoS Genet..

[12] Bukowski M (2013). A regulatory role for Staphylococcus aureus toxin-antitoxin system PemIKSa. Nat Commun..

[13] Fernández-García L (2016). Toxin-antitoxin systems in clinical pathogens. Toxins (Basel)..

[14] Page R, Peti W (2016). Toxin-antitoxin systems in bacterial growth arrest and persistence. Nat Chem Biol..

[15] Lee K-Y, Lee B-J (2016). Structure, biology, and therapeutic application of toxin–antitoxin systems in pathogenic bacteria. Toxins (Basel)..

[16] Pimentel B (2014). Toxin kid uncouples DNA replication and cell division to enforce retention of plasmid R1 in Escherichia coli cells. Proc Natl Acad Sci USA.

[17] Ramage HR (2009). Comprehensive functional analysis of mycobacterium tuberculosis toxin-antitoxin systems: implications for pathogenesis, stress responses, and evolution. PLoS Genet..

[18] Sala A, Bordes P, Genevaux P (2014). Multiple toxin-antitoxin systems in Mycobacterium tuberculosis. Toxins (Basel)..

[19] Georgiades K, Raoult D (2011). Genomes of the most dangerous epidemic bacteria have a virulence repertoire characterized by fewer genes but more toxin-antitoxin modules. PLoS One..

[20] Brown JM, Shaw KJ (2003). A novel family of Escherichia coli toxin-antitoxin gene pairs. J Bacteriol..

[21] Sevin EW, Barloy-Hubler F (2007). RASTA-Bacteria: a web-based tool for identifying toxin-antitoxin loci in prokaryotes. Genome Biol..

[22] Leplae R (2011). Diversity of bacterial type II toxin–antitoxin systems: a comprehensive search and functional analysis of novel families. Nucleic Acids Res..

[23] Sberro H (2013). Discovery of functional toxin/antitoxin systems in bacteria by shotgun cloning. Mol Cell..

[24] Makarova KS, Wolf YI, Koonin EV (2009). Comprehensive comparative-genomic analysis of type 2 toxin-antitoxin systems and related mobile stress response systems in prokaryotes. Biol Direct..

[25] Tong SYC, Davis JS, Eichenberger E, Holland TL, Fowler VG (2015). Staphylococcus aureus infections: epidemiology, pathophysiology, clinical manifestations, and management. Clin Microbiol Rev..

[26] Pires Dos Santos T, Damborg P, Moodley A, Guardabassi L (2016). Systematic review on global epidemiology of methicillin-resistant Staphylococcus pseudintermedius: inference of population structure from multilocus sequence typing data. Front Microbiol..

[27] Yoshizumi S (2009). Staphylococcus aureus YoeB homologues inhibit translation initiation. J Bacteriol..

[28] Schuster CF (2013). Characterization of a mazEF toxin-antitoxin homologue from Staphylococcus equorum. J Bacteriol..

[29] Chanchaithong P, Prapasarakul N, Perreten V, Schwendener S (2016). Characterization of a novel composite staphylococcal cassette chromosome *mec* in methicillin-resistant Staphylococcus pseudintermedius from Thailand. Antimicrob Agents Chemother..

[30] Malachowa N, Deleo FR (2010). Mobile genetic elements of Staphylococcus aureus. Cell Mol Life Sci..

[31] Koreen L (2004). N. spa typing method for discriminating among Staphylococcus aureus isolates: implications for use of a single marker to detect genetic micro- and macrovariation. J Clin Microbiol..

[32] Drancourt M, Raoult D (2002). rpoB gene sequence-based identification of Staphylococcus species. J Clin Microbiol..

[33] Bukowski M (2015). Species determination within Staphylococcus genus by extended PCR-restriction fragment length polymorphism of saoC gene. FEMS Microbiol Lett..

[34] Altschul SF, Gish W, Miller W, Myers EW, Lipman DJ (1990). Basic local alignment search tool. J Mol Biol..

[35] Altschul SF (1997). Gapped BLAST and PS I-BLAST: a new generation of protein database search programs. Nucleic Acids Res..

[36] Altschul SF, Koonin EV (1998). Iterated profile searches with PSI-BLAST—a tool for discovery in protein databases. Trends Biochem Sci..

[37] Boratyn GM (2012). Domain enhanced lookup time accelerated BLAST. Biol Direct..

[38] Henikoff S, Henikoff JG (1997). Embedding strategies for effective use of information from multiple sequence alignments. Protein Sci..

[39] Friedberg I, Kaplan T, Margalit H (2000). Evaluation of PSI-BLAST alignment accuracy in comparison to structural alignments. Protein Sci..

[40] Jones DT, Swindells MB (2002). Getting the most from PSI-BLAST. Trends Biochem Sci..

[41] Kaushik S (2013). Improved detection of remote homologues using cascade PSI-BLAST: influence of neighbouring protein families on sequence coverage. PLoS One..

[42] Wei Y-Q, Bi D-X, Wei D-Q, Ou H-Y (2015). Prediction of type II toxin-antitoxin loci in Klebsiella pneumoniae genome sequences. Interdiscip Sci Comput Life Sci..

[43] Kullik I, Giachino P (1997). The alternative sigma factor sigmaB in Staphylococcus aureus: regulation of the sigB operon in response to growth phase and heat shock. Arch Microbiol..

[44] Donegan NP, Cheung AL (2009). Regulation of the mazEF toxin-antitoxin module in Staphylococcus aureus and its impact on sigB expression. J Bacteriol..

[45] Senn MM (2005). Molecular analysis and organization of the sigmaB operon in Staphylococcus aureus. J Bacteriol..

[46] Schuster C. F. *et al*. The MazEF Toxin-antitoxin system alters the β-Lactam susceptibility of Staphylococcus aureus. Hayes F, editor. *PLoS One*. **10**, e0126118 (2015). 10.1371/journal.pone.0126118 PMC4428803 25965381

[47] Chan PF, Foster SJ, Ingham E, Clements MO (1998). The Staphylococcus aureus alternative sigma factor sigma B controls the environmental stress response but not starvation survival or pathogenicity in a mouse abscess model. J Bacteriol..

[48] Christensen-Dalsgaard M, Gerdes K (2006). Two higBA loci in the Vibrio cholerae superintegron encode mRNA cleaving enzymes and can stabilize plasmids. Mol Microbiol..

[49] Szekeres S, Dauti M, Wilde C, Mazel D, Rowe-Magnus DA (2007). Chromosomal toxin-antitoxin loci can diminish large-scale genome reductions in the absence of selection. Mol Microbiol..

[50] Harrison PW, Lower RPJ, Kim NKD, Young JPW (2010). Introducing the bacterial “chromid”: not a chromosome, not a plasmid. Trends Microbiol..

[51] Flannagan SE (2003). Plasmid content of a vancomycin-resistant Enterococcus faecalis isolate from a patient also colonized by Staphylococcus aureus with a VanA phenotype. Antimicrob Agents Chemother..

[52] de Niederhäusern S (2011). Vancomycin-resistance transferability from VanA Enterococci to Staphylococcus aureus. Curr Microbiol..

[53] Lou C, Li Z, Ouyang Q (2008). A molecular model for persister in E. coli. J Theor Biol..

[54] Helaine S (2014). Internalization of Salmonella by macrophages induces formation of nonreplicating persisters. Science..

[55] Maisonneuve E, Gerdes K (2014). Molecular mechanisms underlying bacterial persisters. Cell..

[56] Zhang Y (2014). Persisters., persistent infections and the Yin-Yang model. Emerg. Microbes Infect..

[57] Fasani RA, Savageau MA (2015). Unrelated toxin-antitoxin systems cooperate to induce persistence. J R Soc Interface..

[58] Zhang Y (2003). MazF cleaves cellular mRNAs specifically at ACA to block protein synthesis in Escherichia coli. Mol Cell..

[59] Rothenbacher FP (2012). Clostridium difficile MazF toxin exhibits selective, not global, mRNA cleavage. J Bacteriol..

[60] Yamaguchi Y, Nariya H, Park JH, Inouye M (2012). Inhibition of specific gene expressions by protein-mediated mRNA interference. Nat Commun..

[61] Park JH, Yamaguchi Y, Inouye M (2011). Bacillus subtilis MazF‐bs (EndoA) is a UACAU‐specific mRNA interferase. FEBS Lett..

[62] Simanshu DK, Yamaguchi Y, Park JH, Inouye M, Patel DJ (2013). Structural basis of mRNA recognition and cleavage by toxin MazF and its regulation by antitoxin MazE in Bacillus subtilis. Mol Cell..

[63] Zorzini V (2016). Substrate recognition and activity regulation of the Escherichia coli mRNA endonuclease MazF. J Biol Chem..

[64] Lowder BV (2009). Recent human-to-poultry host jump, adaptation, and pandemic spread of Staphylococcus aureus. Proc Natl Acad Sci USA..

[65] Bacterial Genomes at GenBank Database. National Center for Biotechnology Information. 2016. ftp://ftp.ncbi.nlm.nih.gov/genomes/genbank/bacteria/. Accessed 2016 Jul 20. (2016).

[66] Camacho C (2009). BLAST+: architecture and applications. BMC Bioinformatics..

[67] Codon Tables. National Center for Biotechnology Information. 2016. https://www.ncbi.nlm.nih.gov/Taxonomy/Utils/wprintgc.cgi. Accessed 2016 Dec 17. (2016).

[68] Marchler-Bauer A (2003). CDD: a curated Entrez database of conserved domain alignments. Nucleic Acids Res..

[69] Barrett T, Clark K, Gevorgyan R, Gorelenkov V, Gribov E, Karsch-Mizrachi I (2012). BioProject and BioSample databases at NCBI: facilitating capture and organization of metadata. Nucleic Acids Res..

[70] BioSample Database. National Center for Biotechnology Information. 2016. https://www.ncbi.nlm.nih.gov/biosample. Accessed 2016 Dec 17.

[71] Maglott D, Ostell J, Pruitt KD, Tatusova T (2005). Entrez Gene: gene-centered information at NCBI. Nucleic Acids Res..

[72] Wheeler DL (2005). Database resources of the National Center for Biotechnology Information. Nucleic Acids Res..

[73] Polakowska K (2012). The virulence of Staphylococcus aureus correlates with strain genotype in a chicken embryo model but not a nematode model. Microbes Infect..

