# Supplementary material for: Identification of novel mazEF/pemIK family toxin-antitoxin loci and their distribution in the Staphylococcus genus
Source: Sci Rep. 2017 Oct 18;7:13462. doi: 10.1038/s41598-017-13857-4 (PMC5647390; doi:10.1038/s41598-017-13857-4)
Supplement: Supplementary file 1 — Supplementary data [file 41598_2017_13857_MOESM1_ESM.pdf]

Identification of novel *mazEF/pemIK* family toxin-antitoxin loci and their distribution in the *Staphylococcus* genus

Michal Bukowski[1], Karolina Hyz[1], Monika Janczak[1], Marcin Hydzik[1], Grzegorz Dubin[2,3], Benedykt Wladyka[1,#]

[1]Department of Analytical Biochemistry, Faculty of Biochemistry, Biophysics and Biotechnology, Jagiellonian University, Krakow, Poland
[2]Malopolska Centre of Biotechnology, Jagiellonian University, Krakow, Poland
[3]Department of Microbiology, Faculty of Biochemistry, Biophysics and Biotechnology, Jagiellonian University, Krakow, Poland

#Corresponding author:
Benedykt Wladyka
Department of Analytical Biochemistry
Faculty of Biochemistry, Biophysics and Biotechnology
Jagiellonian University
7 Gronostajowa St, 30-387 Krakow, Poland
phone: +48 12 664 65 11
fax: +48 12 664 69 15
e-mail: benedykt.wladyka@uj.edu.pl

Keywords: *mazEF*, mRNA interferase, *pemIK*, staphylococci, *Staphylococcus*, *Staphylococcus aureus*, TA system, toxin-antitoxin system

SUPPLEMENTARY DATA

Supplementary note on construction of phylogenetic trees…….. 2
Supplementary figure 1…………………………………………….. 3
Supplementary figure 2…………………………………………….. 4
Supplementary figure 3…………………………………………….. 5
Supplementary figure 4…………………………………………….. 6
Supplementary figure 5…………………………………………….. 7
Supplementary figure 6…………………………………………….. 8
Bibliography………………………………………………………… 9

Supplementary note on construction of phylogenetic trees. The following parameters were used for multiple sequence alignments of DNA sequences; gap open cost: 20.0; gap extension cost: 1.0; end gap cost: free; alignment mode: very accurate; and the following ones for protein sequences; gap open cost: 10.0; gap extension cost: 1.0; end gap cost: free; alignment mode: very accurate. For DNA sequences the model choice and parameters were as following; construction method: neighbour joining [1]; nucleotide substitution model: general time reversible, GTR [2]; transition/transversion ratio: 2.0; include rate variation: yes; number of substitution rate categories: 8; gamma distribution parameter: 1.0; estimate substitution rate parameters, topology and gamma distribution parameter: yes; bootstrap analysis with 100 replicates; and for protein sequences as following; construction method: neighbour joining [1]; protein substitution model: WAG [3]; transition/transversion ratio: 2.0; include rate variation: yes; number of substitution rate categories: 8; gamma distribution parameter: 1.0; estimate substitution rate parameters, topology and gamma distribution parameter: yes; bootstrap analysis with 100 replicates. Prototypical protein sequences of all MazF/PemK toxins were aligned with the following parameters: gap open cost: 20.0; gap extension cost: 1.0; end gap cost: as any other; alignment mode: very accurate; and visualised using CLC Main Workbench.

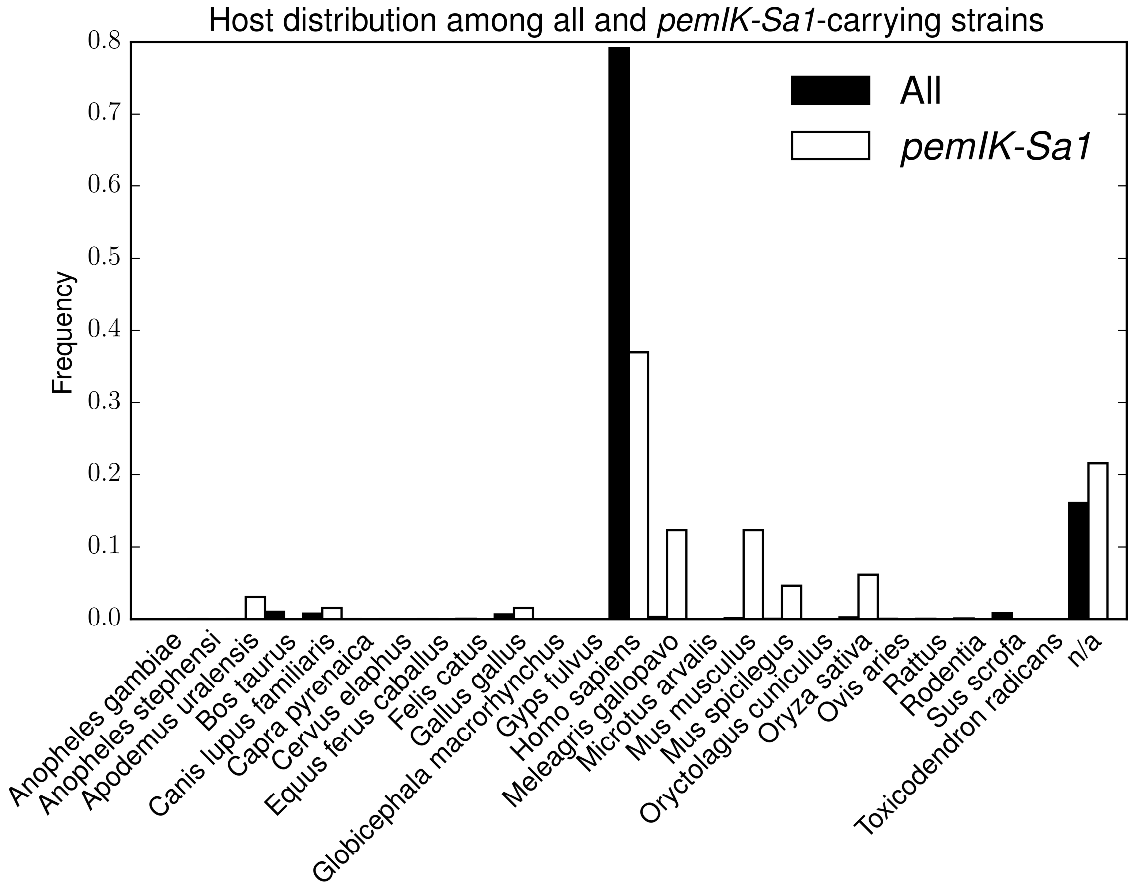

Suppl. fig. 1. Host distribution of *pemIK-Sa1* loci.

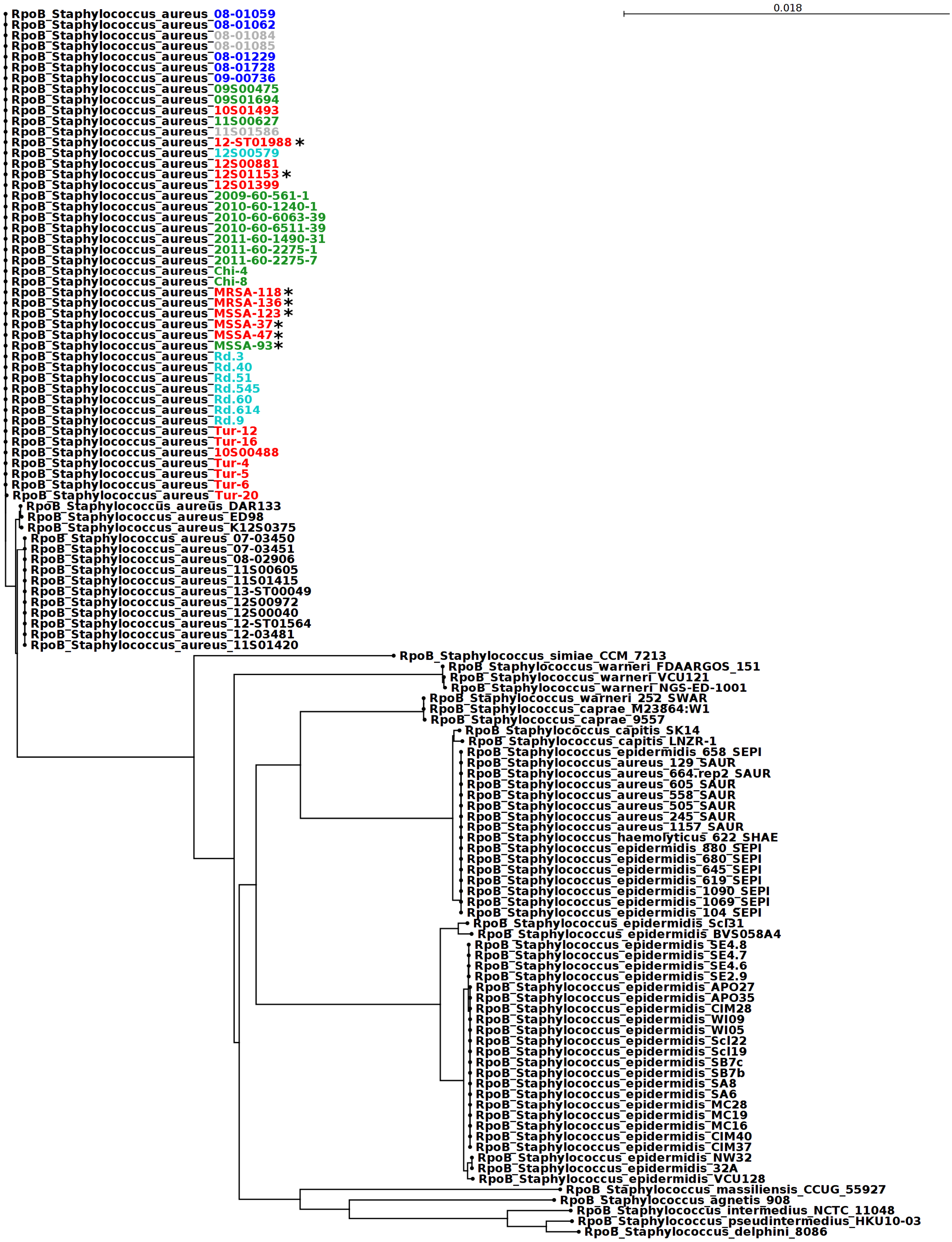

Suppl. fig. 2. Phylogenetic tree based on gapless alignment of *rpoB* gene sequences derived from strains isolated in Germany and all strains possessing *pemIK-Sa1* locus. Clolours of signatures of closely related strainsdenote the host:: unknown, light grey; *Bos taurus*, skyblue; *Gallus gallus*, green; *Homo sapiens*, blue; *Meleagris gallopavo*, red. The *pemIK-Sa1*-carrying strains are marked with asterisk.

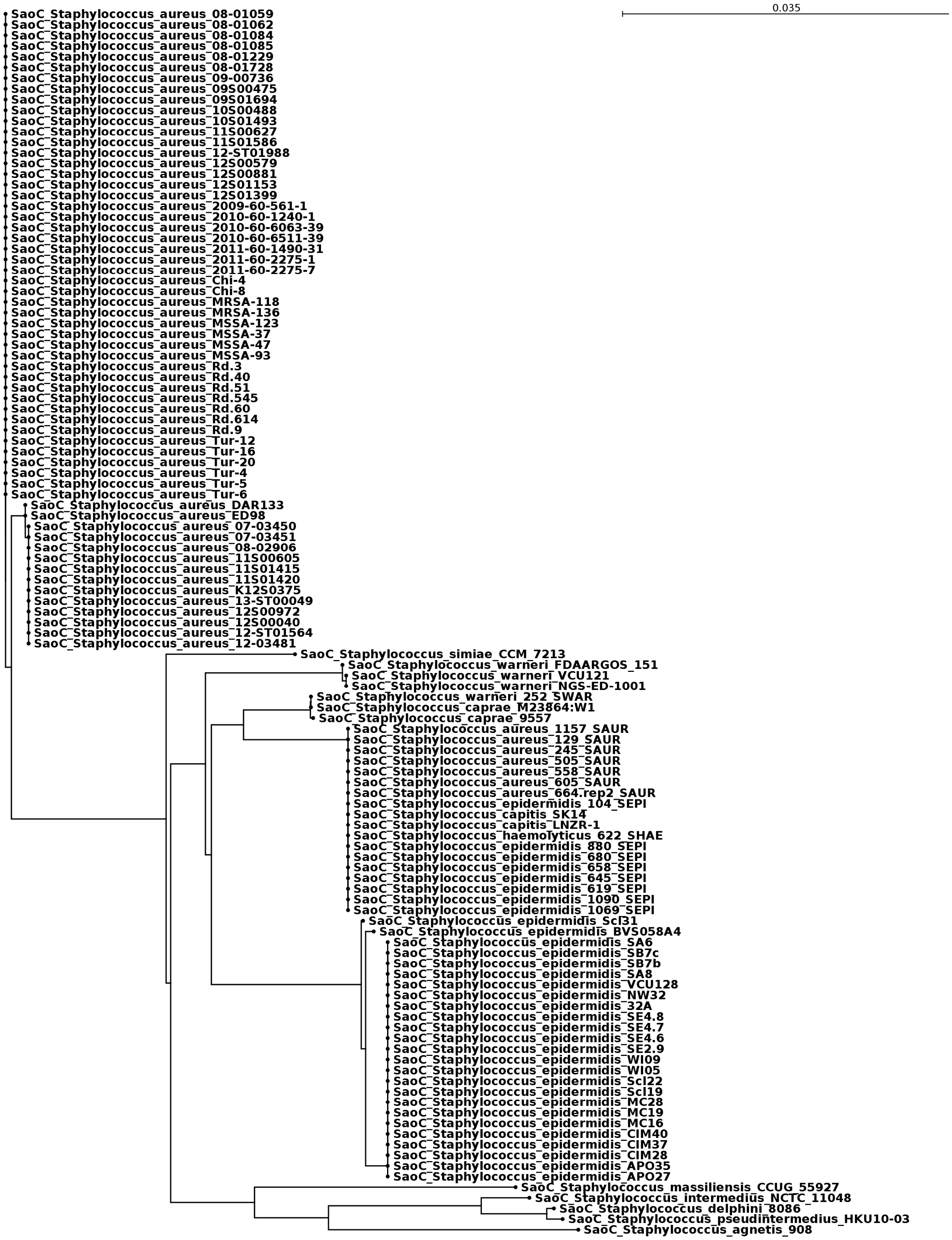

Suppl. fig. 3. Phylogenetic tree based on gapless alignment of *saoC* gene sequences derived from strains isolated in Germany and all strains possessing *pemIK-Sa1* locus.

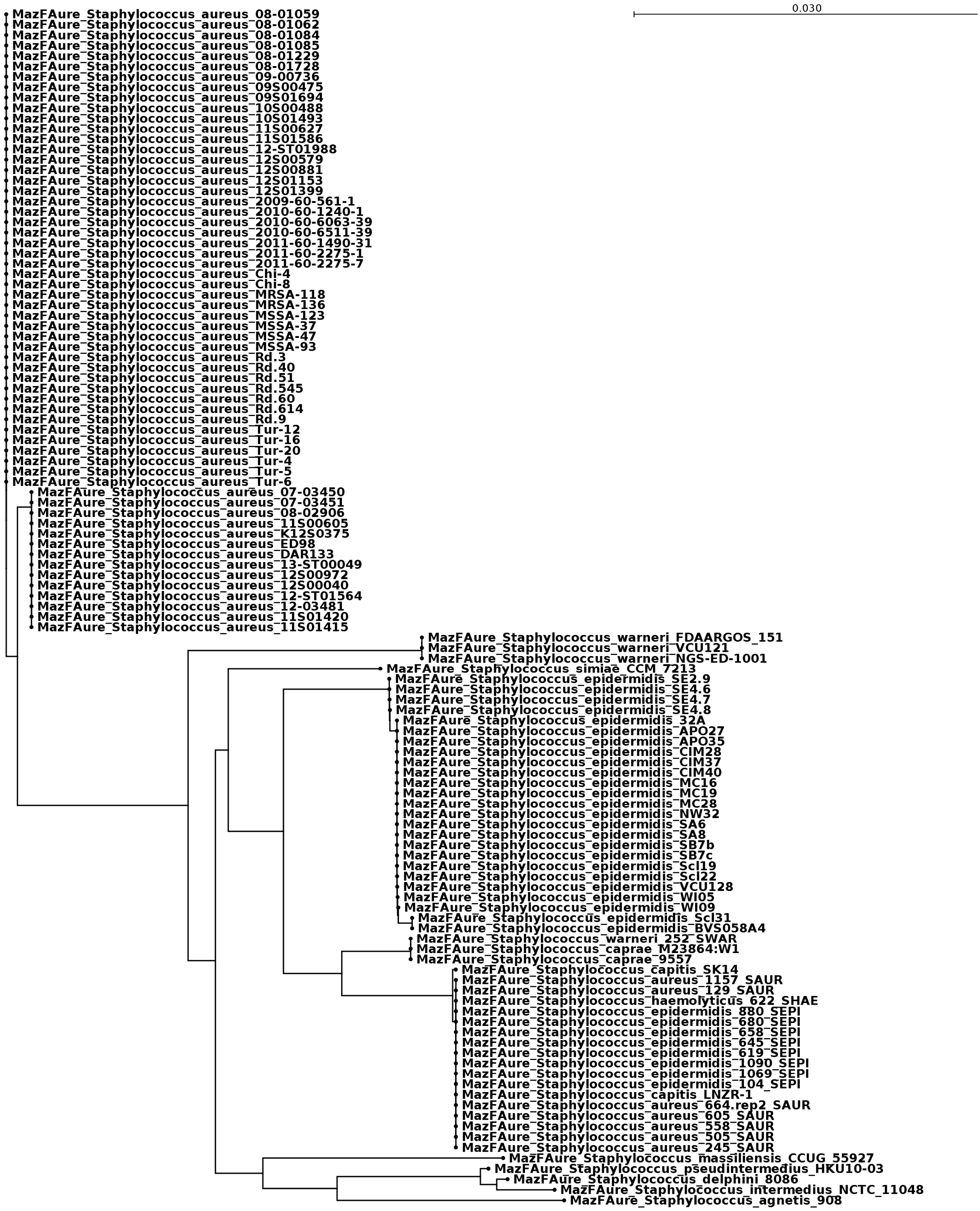

Suppl. fig. 4. Phylogenetic tree based on gapless alignment of *mazF* gene sequences derived from strains isolated in Germany and all strains possessing *pemIK-Sa1* locus.

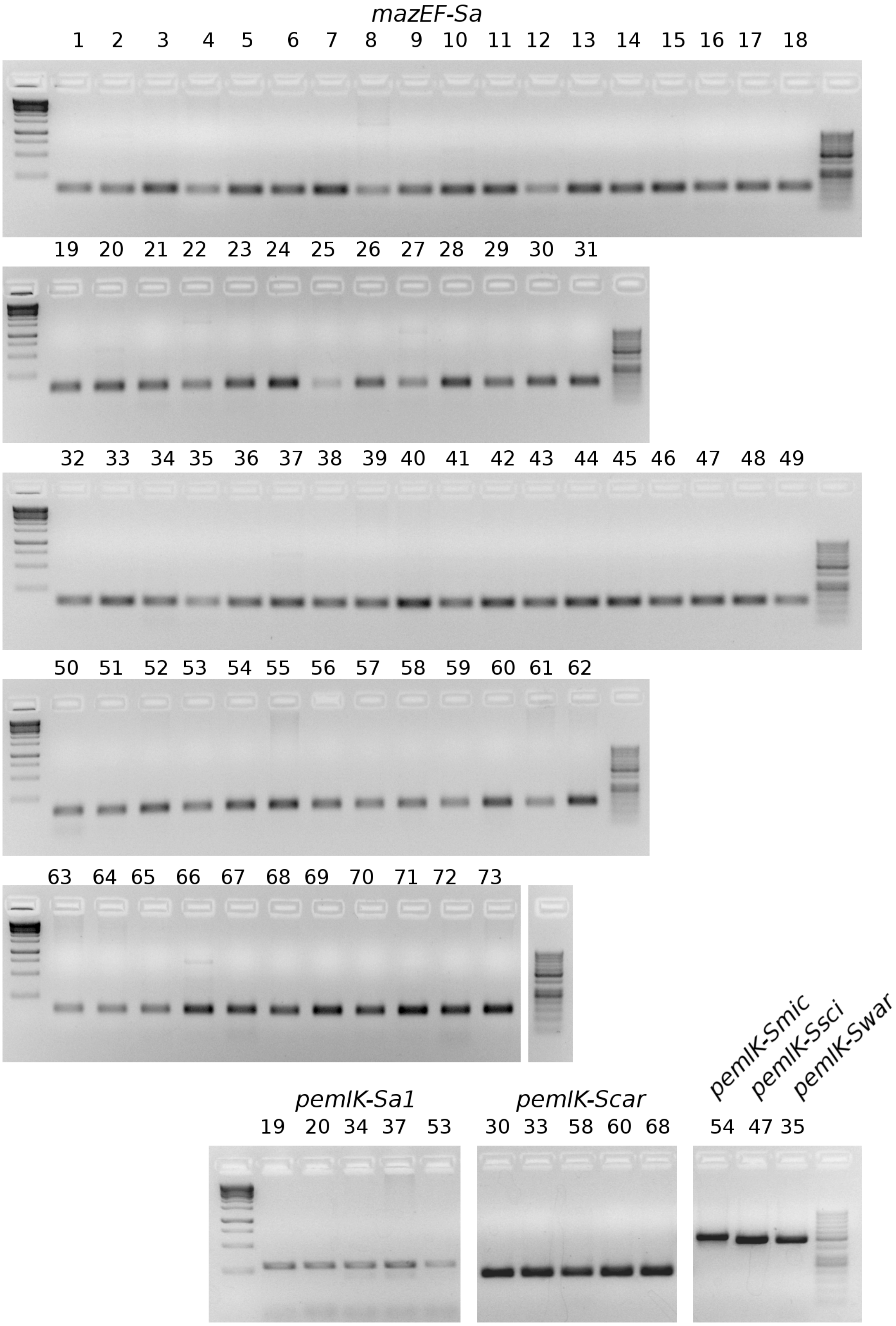

Suppl. fig. 5. Whole-lane images used for preparation of fig. 10: PCR screen for *mazEF/pemIK* loci. The numbers correspond to the number in fig. 10. The first and the last lane: length standards, respectively Gene Ruler 1 kb and Gene Ruler 50 bp (Thermocientific). White gaps: irrelevant lanes were removed. All fragments in one row are fragments of the same gel image.

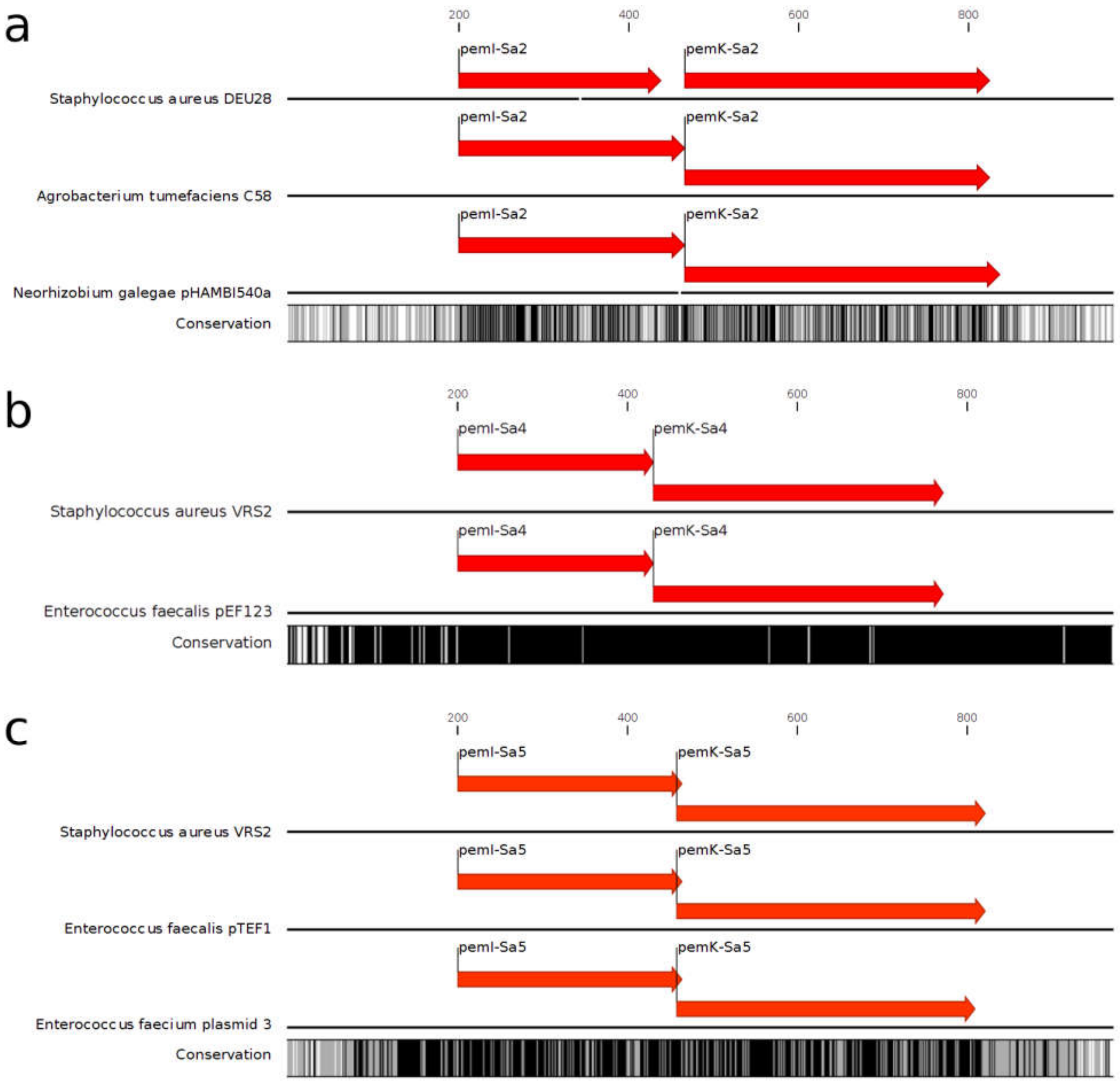

Suppl. fig. 6. Conservation of nucleotide sequences of different *pemIK* loci occurring in *S. aureus* and other species. The conservation scale ranges from 50% to 100% (white to black). Each fragment is ~ 1 kb long, the bp ruler is placed above each alignment. In all cases higher conservation is observed within the coding sequences, which when translated are more than 80% similar. The adjacent non-coding sequences are more diverse, likely due to variability in species-specific promoter and other regulatory sequences. (**a**) *pemIK-Sa2* loci are the most divergent, still much higher conservation of coding sequences is clearly visible. (**b**) On the other hand, although *pemIK-Sa4* sequences are highly conserved, there are clear differences in 5'-UTRs. (**c**) *pemIK-Sa5* loci are more diverse than co-occurring with them *pemIK-Sa4* ones and as in other cases higher variability in non-conding sequences is observed.

Bibliography

1. Saitou N, Nei M. The neighbor-joining method: a new method for reconstructing phylogenetic trees. Mol Biol Evol. 1987;4(4):406–25.

2. Nei M, Kumar S. Molecular Evolution and Phylogenetics. Oxford University Press; 2000.

3. Whelan S, Goldman N. A general empirical model of protein evolution derived from multiple protein families using a maximum-likelihood approach. Mol Biol Evol. 2001;18(5):691–9.
